# Targeted mutation screening of 292 candidate genes in 38 children with inborn haematological cytopenias efficiently identifies novel disease‐causing mutations

**DOI:** 10.1111/bjh.15389

**Published:** 2018-05-24

**Authors:** Leo Kager, Raúl Jimenez Heredia, Tatjana Hirschmugl, Jasmin Dmytrus, Ana Krolo, Heiko Müller, Christoph Bock, Petra Zeitlhofer, Michael Dworzak, Georg Mann, Wolfgang Holter, Oskar Haas, Kaan Boztug

**Affiliations:** ^1^ St Anna Children's Hospital Department of Paediatrics and Adolescent Medicine Medical University of Vienna Vienna Austria; ^2^ Children's Cancer Research Institute Vienna Austria; ^3^ Ludwig Boltzmann Institute for Rare and Undiagnosed Diseases Vienna Austria; ^4^ CeMM Research Centre for Molecular Medicine of the Austrian Academy of Sciences Vienna Austria; ^5^ medgen.at GmbH Vienna Austria

**Keywords:** clinical haematology, immunodeficiency, paediatric haematology, genetic disorders

## Abstract

Establishing a precise diagnosis is essential in inborn haematological cytopenias to enable appropriate treatment decisions and avoid secondary organ damage. However, both diversity and phenotypic overlap of distinct disease entities may make the identification of underlying genetic aetiologies by classical Sanger sequencing challenging. Instead of exome sequencing, we established a systematic next generation sequencing‐based panel targeting 292 candidate genes and screened 38 consecutive patients for disease‐associated mutations. Efficient identification of the underlying genetic cause in 17 patients (44·7%), including 13 novel mutations, demonstrates that this approach is time‐ and cost‐efficient, enabling optimal management and genetic counselling.

## Introduction

Haematological cytopenias, i.e., deficiencies of one or several blood cell components, are the shared hallmark of an extremely heterogeneous group of either environmentally or genetically triggered diseases. The identification of the underlying genetic aetiology of these diseases is particularly important in children, in whom inborn disorders predominate. These disorders comprise red blood cell membrano‐ and enzymopathies, bone marrow failure syndromes, familial haemophagocytic lymphohistiocytosis, mitochondriopathies as well as primary immunodeficiencies. Except for haemoglobinopathies and hereditary spherocytosis, all of these are orphan or even ultra‐orphan diseases.

**Figure 1:**
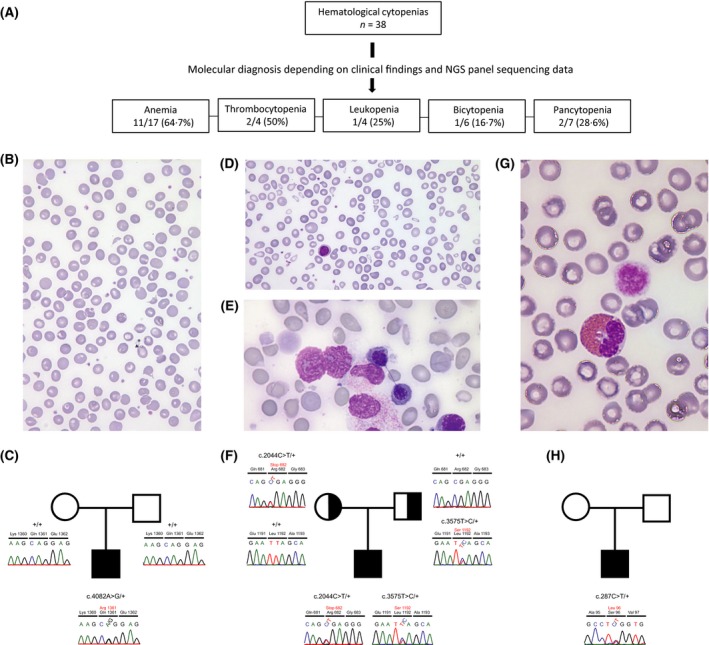
Targeted NGS‐based panel screen for efficient identification of genetic causes in inherited haematological diseases. (A) A total of 38 patients with molecularly undefined cytopenias were included in the study, and a disease‐causing mutation was identified in 17 (44·7%). (B) May‐Grünwald‐Giemsa stained peripheral blood smear showing characteristic dessicytes and a few target cells in Patient 8 with hereditary xerocytosis caused by (C) a novel mutation in *PIEZO1* (NM_001142864.2:c.4082A>G p.Q1361R). In Patient 9, who exhibited characteristic light microscopy findings in (D) peripheral blood, i.e., gross anisocytosis, poikilocytosis and (E) bone marrow, such as erythroid hyperplasia and chromatin bridges between nuclei of two separate erythroblasts, we identified (F) novel compound heterozygous variants (NM_138477.2:c.2044C>T p.R682X and NM_138477.2:c.3575T>C p.L1192S) in codanin 1 (*CDAN1*) causing congenital dyserythropoietic anaemia type 1. In an infant (Patient 13) with thrombocytopenia and giant platelets (G) [giant platelet (right) and eosinophilic granulocyte (left)], we identified a (H) known causative mutation in *MHY9* (NM_002473.4:c.287C>T p.S96L) causing MYH9‐related disease. NGS, next generation sequencing.

Our study was approved by the responsible institutional review board and performed with the written informed consent from patients, parents and/or legal guardians. We included 38 children and young adults (17 males and 21 females) with a median age of 7·5 years (range 0·1–21·4 years; Fig 1A), who were referred consecutively to our hospital and who fulfilled established cytopenia criteria. The patients’ DNA was analysed with a custom‐designed targeted enrichment panel (HaloPlex™; Agilent Technologies, Santa Clara, CA, USA) that comprised the respective 292 candidate genes (Table S1) similar to a recently established targeted panel for genetic investigation of primary immunodeficiencies (Erman *et al*, 2017). Regions of interest included all exons, exon–intron boundaries and promoter regions. Sequencing was performed on a HiSeq3000 platform (Illumina, San Diego, CA, USA) as described (Salzer *et al*, 2016; Erman *et al*, 2017). 98·5% of enriched exonic bases were considered callable with a minimum read depth of 2. The same calculation for a minimum read depth of 10 and 40, revealed 96·4% and 88·2% coverage, respectively (not shown). Sequencing data were analysed with a previously established next generation sequencing (NGS)‐data analysis pipeline that is based on current Genome Analysis Toolkit (GATK) best practice recommendations (Salzer *et al*, 2016; Erman *et al*, 2017). The potential relevance and recurrence of variants was assessed with prediction tools (e.g. CADD), ExAC and our internal dataset of more than 400 sequenced individuals, as described (Salzer *et al*, 2016). Quality metrics were generated using CollectHsMetrics (http://broadinstitute.github.io/picard/command-line-overview.html, last accessed 13 April 2017) for analysis of target‐capture sequencing data. Additional details and genetic results are outlined in the Supplementary Results. All identified variants which were deemed potentially disease‐causing were validated with capillary sequencing as described (Salzer *et al*, 2016; Erman *et al*, 2017) or multiplex ligation‐dependent probe amplification.

Collectively, we identified a causative genetic defect in 17/38 patients (44·7%) including novel mutations in 13 of them (Fig 1A). In 8/17 (47%) patients the results of molecular testing were considered as ‘confirmatory’ and ‘diagnostic’ in 9/17 (53%), respectively. (Table 1). Eleven of the patients presented with anaemia (clinical and haematological details are provided in Table S2) five of them with a normocytic (spherocytic), chronic, haemolytic anaemia (CHA; Patients 1–5; Table 1) and six with a macrocytic anaemia (Patients 6–11, Table 1). Within the subgroup of five patients with hereditary spherocytosis (HS), we identified heterozygous mutations in the Spectrin Beta, Erythrocytic gene (*SPTB*) in two, and mutations in the *Ankyrin 1* (*ANK1*) membrane protein encoding gene in the remaining three patients (Table 1). Contrary to the originally suspected congenital dyserythropoietic anaemia (CDA), Patient 6 had a pyruvate kinase deficient anaemia due to a homozygous *PKLR* mutation (Table 1) (Unal & Gumruk, 2015). Two patients with mild macrocytic anaemia had mutations in the gene encoding the piezo‐type mechanosensitive ion channel component 1 (*PIEZO1*)*:* Patient 7 bore a previously described pathogenic p.R2456H variant (Zarychanski *et al*, 2012), and Patient 8 a novel p.Q1361R mutation located adjacent to the already known p.R1358P mutation (Table 1) (Albuisson *et al*, 2013). This finding, together with the subsequent perception of dessicytes in the respective blood smears (Fig 1B,C) was particularly relevant, given that splenectomy had been considered prior to identifying the disease as hereditary xerocytosis (HX); this form of treatment is contraindicated in HX because of an associated inherent increased risk of thromboembolic complications (Andolfo *et al*, 2016). In Patients 9 and 10, novel compound heterozygous mutations in codanin 1 (*CDAN1*) confirmed the respective diagnosis of CDA type 1 (CDA1), which originally had solely been based on the morphological abnormalities of erythropoietic components in the peripheral blood and bone marrow (Fig 1D–F; Table 1). Furthermore, we also identified a novel mutation in the ribosomal protein S29 gene (*RPS29*, p.A47T) in patient 11 with Diamond‐Blackfan anaemia (DBA) (Table 1).

**Table 1:** Identification of causative genetic variants in 11 patients with inherited anaemias using targeted NGS‐based panel sequencing

Patient	Age (years)	Sex	Key clinical features	Key haematological features	Gene	Mutation	Type of mutation	Inheri‐tance	Final Diagnosis	Clinical Relevance	Additional information
1	13·7	M	Jaundice, splenomegaly, gall stones	Moderate normocytic CHA with spherocytes	*SPTB*	NM_001024858.2:c.5961_5964delinsTTC p.M1988Sfs*7	Het, novel	AD	Hereditary spherocytosis	Confirmatory	Reduced EMA staining *UGT1A1* wild‐type
2	8	F	Jaundice, splenomegaly, gall stones	Moderate normocytic CHA with spherocytes	*SPTB*	NM_001024858.2:c.4309dupG; p.E1437Gfs*54	Het, novel	AD	Hereditary spherocytosis	Confirmatory	Reduced EMA staining, subtotal splenectomy
3	13·6	M	Jaundice, splenomegaly, gall stones	Moderate normocytic CHA with spherocytes	*ANK1*	NM_001142446.1:c.457C>T p.Q153*	Het, novel	AD	Hereditary spherocytosis	Confirmatory	Reduced osmotic fragility, cholecystectomy + subtotal splenectomy at 5 years, spleen regrowth. *UGT1A1* wild‐type
4	1	M	Jaundice	Moderate normocytic CHA with spherocytes	*ANK1*	NM_001142446.1:c.4510_4513delAACA p.N1504Wfs*17	Het, novel	AD	Hereditary spherocytosis	Confirmatory	Reduced EMA staining, transfusions until age 7 months
5	2·7	F	Jaundice	Moderate normocytic CHA with spherocytes	*ANK1*	NM_001142446.1:c.1872_1884delGGGCGGCTCCCCG p.G625Tfs*41	Het, novel	AD	Hereditary spherocytosis	Confirmatory	Reduced EMA staining
6	4·6	F	Jaundice, splenomegaly	Severe macrocytic CHA, dyserythropoiesis	*PKLR*	NM_000298.5:c.1675C>G p.R559G	Hom	AR, Con	Pyruvate kinase deficiency	Diagnostic, recommend splenectomy	PK activity low normal range, parents normal PK activity
7	19·2	M	Jaundice, splenomegaly, gall stones	Mild macrocytic CHA, dessicytes	*PIEZO1*	NM_001142864.2:c.7367G>A p.R2456H	Het	AD	Hereditary xerocytosis	Diagnostic, avoid splenectomy	Admitted with diagnosis of ‘familial hyperbilirubinaemia’
8	10·1	F	Jaundice, splenomegaly	Mild macrocytic CHA, dessicytes, dyserythropoiesis	*PIEZO1*	NM_001142864.2:c.4082A>G p.Q1361R	Het, novel	AD, *de novo*	Hereditary xerocytosis	Diagnostic, avoid splenectomy	*UGT1A1* promotor variant aggravates jaundice
9	3·4	M	Splenomegaly	Severe macrocytic CHA, aniso‐poikilocytes; dyserythropoiesis, inter‐nuclear bridges	*CDAN1*	NM_138477.2:c.2044C>T p.R682* c.3575T>C p.L1192S†	Com, †novel	AR	CDA1	Diagnostic, consider interferon therapy	Transfusion dependent as infant, later on moderate anaemia
10	17·9	M	Jaundice, cardiac defects, skeletal defects	Moderate macrocytic CHA, aniso‐poikilocytes; dyserythropoiesis inter‐nuclear bridges	*CDAN1*	NM_138477.2:c.2015C>T p.P672L† c.1189C>T p.R397W	Com, †novel	AR	CDA1	Diagnostic, consider interferon therapy	Extramedullary haematopoiesis of the skull, Chiari 1 malformation, syringomyelia, surgery for cardiac defects in infancy
11	1·2	M	Paleness, fatigue	Severe macrocytic hyporegenerative anaemia, lack of BM erythroblasts	*RPS29*	NM_001032.4:c.139G>A p.A47T	Het, novel	AD	Diamond‐Blackfan anaemia	Confirmatory, offer steroid therapy	Elevated erythrocyte adenosine deaminase and HbF

AD, autosomal dominant; *ANK1*, ankyrin 1; AR, autosomal recessive; BM, bone marrow; CDA1, congenital dyserythro‐poietic anaemia type I; CDAN1, codanin 1; CHA, chronic haemolytic anaemia; Com, compound heterozygous; Con, consanguineous; EMA, eosin‐5′‐maleimide: F, female, *FANCA*, Fanconi anaemia complementation group A; *GATA2*, GATA binding protein 2; HbF, haemoglobin F; Het, heterozygous; Hom, homozygous; M, male, MDS, myelodysplastic syndrome; *MYH9*, myosin heavy chain 9; NGS, next generation sequencing; *NHEJ1*, non‐homologous end joining factor 1; *PIEZO1*, piezo type mechanosensitive ion channel component 1; PK, pyruvate kinase; *PKLR*, pyruvate kinase, liver and RBC; *RPL5*, ribosomal protein L5; *RPS29*, ribosomal protein S29; *RUNX1*, runt related transcription factor 1; *SPTB*, spectrin beta, erythrocytic; *UGT1A1*, UDP glucuronosyltransferase family 1 member A1. *, Stop codon. †, novel mutation in compound heterozygous cases.

Disease‐relevant mutations were discovered in two of four patients with thrombocytopenia. The severe form of macro‐thrombocytopenia in an infant (Patient 13; Table 2; Fig 1G,H; Table S3), was the consequence of a heterozygous mutation in the gene encoding the myosin heavy chain 9 (*MYH9*, p.S96L), while a novel heterozygous p.G165R mutation in the *RUNX1* gene, which encodes the haematopoietic runt related transcription factor 1, was responsible for a mild form of chronic normocytic thrombocytopenia in an eight‐year‐old girl (Patient 12; Table 2; Table S3) (Sood *et al*, 2017).

**Table 2:** Identification of causative genetic variants in 6 patients with inherited cytopenias other than anaemias using targeted NGS‐based panel sequencing

Patient	Type	Age (years)	Sex	Key clinical features	Key haematological features	Gene	Mutation	Type of mutation	Inheri‐tance	Final Diagnosis	Clinical relevance	Additional information
12	Thrombo‐cytopenia	8·8	F	None	Mild thrombocytopenia	*RUNX1*	NM_001754.4:c.493G>C p.G165R	Het, novel	AD	RUNX1‐associated thrombocytopenia	Diagnostic, impacts management	Family history inconspicuous
13	Thrombo‐cytopenia	0·9	M	Bruising	Severe macrocytic thrombocytopenia (giant platelets)	*MYH9*	NM_002473.4:c.287C>T p.S96L	Het	AD	MYH9‐related disorder	Diagnostic, impacts management	No hearing or renal pathologies yet
14	Leucopenia	17·8	F	Recurrent fever and aphthous lesions	Moderate neutropenia and B cell deficiency	*GATA2*	NM_032638.4:c.121C>G p.P41A	Het	AD	GATA2 deficiency	Diagnostic, impacts management	One episode of severe pancytopenia
15	Bicytopenia	13	M	Growth retardation, facial defects, cardiac defects	Severe hyporegenerative macrocytic anaemia, moderate leucopenia	*RPL5*	NM_000969.3:c.527 + 2dupT p.?	Het, novel	AD	Diamond‐Blackfan anaemia	Confirmatory	Steroid responder, but currently steroid pause (puberty); regular transfusions
16	Pancytopenia	8·3	F	Broad nasal base and epicanthic folds, cardiac defect	Moderate thrombocytopenia, mild leucopenia and mild macrocytic anaemia	*FANCA*	Deletion of Exons 6–31	Hom, novel	AR	Fanconi Anaemia	Confirmatory, impacts management	Twin sister has same variant and clinical presentation
17	Pancytopenia	21·4	M	Microcephaly, growth retardation, skeletal anomalies	Mild pancytopenia, mild T‐lymphopenia and severe B‐lymphopenia; MDS	*NHEJ1*	NM_024782.2:c.236T>C p.L79P	Hom, novel	AR	Combined immunodeficiency with MDS	Diagnostic, impacts management	MDS; initially monosomy 7, replaced by del(20)

AD, autosomal dominant; *ANK1*, ankyrin 1; AR, autosomal recessive; BM, bone marrow; CDA1, congenital dyserythro‐poietic anaemia type I; CDAN1, codanin 1; CHA, chronic haemolytic anaemia; Com, compound heterozygous; Con, consanguineous; EMA, eosin‐5′‐maleimide: F, female, *FANCA*, Fanconi anaemia complementation group A; *GATA2*, GATA binding protein 2; HbF, haemoglobin F; Het, heterozygous; Hom, homozygous; M, male, MDS, myelodysplastic syndrome; *MYH9*, myosin heavy chain 9; NGS, next generation sequencing; *NHEJ1*, non‐homologous end joining factor 1; *PIEZO1*, piezo type mechanosensitive ion channel component 1; PK, pyruvate kinase; *PKLR*, pyruvate kinase, liver and RBC; *RPL5*, ribosomal protein L5; *RPS29*, ribosomal protein S29; *RUNX1*, runt related transcription factor 1; *SPTB*, spectrin beta, erythrocytic; *UGT1A1*, UDP glucuronosyltransferase family 1 member A1.

Of the four leucopenic patients, the only mutation detected was in a 17‐year‐old female with moderate neutropenia and B cell deficiency, who suffered from recurrent fever bouts and oral aphthous lesions (Patient 14: Table 2; Table S4). Her phenotype can probably be explained by a heterozygous variant (p.P41A) in the gene encoding the haematopoietic transcription factor GATA binding protein 2 (*GATA2*), which had been documented previously in a patient with myelodysplastic syndrome (MDS) (Holme *et al*, 2012), despite a frequency of heterozygotes of 0·21% (134 of 123 458 total alleles) in gnomAD (http://gnomad.broadinstitute.org/, accessed March 2018) amongst non‐Finnish Europeans (Table 2).

Similarly, we only discovered a clear genetic disease cause in one of seven patients with bicytopenia. He suffered from steroid‐responsive DBA with anaemia and leucopenia and carried a novel heterozygous splice‐site donor frame shift mutation in the ribosomal protein *RPL5* gene (Patient 15; Table 2; Table S5).

We also discovered disease‐predisposing mutations in two of seven patients with pancytopenia (clinical details of the two patients are provided in Table S6). We identified a unique deletion encompassing exons 6–31 of the *FANCA* gene in an eight‐year‐old girl with Fanconi anaemia (Patient 16; Table 2; Figure S1) and a novel mutation in the non‐homologous end joining factor 1 gene (*NHEJ1*, also termed *Cernunnos*) (Patient 17; Table 2). This finding not only disclosed that he suffered from a special form of immunodeficiency with a severe B cell deficiency, but also helped to explain his physical problems, growth retardation, microcephaly and clinodactyly, which are also salient features of this syndrome (Buck *et al*, 2006). He developed a mild pancytopenic form of MDS with monosomy 7, which was eventually replaced by a 20q deletion.

As an alternative approach to whole exome or genome sequencing (Yang *et al*, 2013), targeted sequencing panels have hitherto been used for more narrowly defined groups of haematological disorders, such as inherited anaemias (Agarwal *et al*, 2016; Roy *et al*, 2016), inherited bone marrow failure syndromes (Ghemlas *et al*, 2015), DBA (Gerrard *et al*, 2013) and thrombocytopathies (Lentaigne *et al*, 2016). Given both the phenotypic overlap and diverse genotypic spectrum of many of these conditions, we deliberately chose to use an extended gene panel that covered a broad spectrum of inborn haematological diseases. This approach enabled us to include patients with diverse haematological conditions and thereby to also discover uncommon and unexpected diseases, such as HX (Patients 7 and 8), CDA1 (Patients 9 and 10), RUNX1‐related thrombocytopenia (Patient 12), GATA2‐related neutropenia (Patient 14), or NHEJ‐related combined immunodeficiency with transformation to MDS (Patient 17). Our overall diagnostic success rate of 44·7% is therefore higher than similar targeted sequencing panel studies performed previously. Given that 13 of our 17 genetically identified cases carried novel mutations and that these also included several cases with ultra‐rare haematological diseases, of which only few cases are known so far, suggests that many such cases may remain unrecognized and undiagnosed at tertiary referral centres.

Considering the inherent difficulties often experienced with the conventional diagnostic evaluation of childhood cytopenias, their prevalent genetic origin, the plethora of potentially involved genes and the heterogeneous types and distribution of mutations, together with the reassuring results presented herein strongly argue for the implementation of such targeted sequencing screening programmes in the initial diagnostic work‐up of such diseases.

## Author contribution

L.K. and K.B. conceived and designed the study; L.K., R.J.H., T.H., J.D., A.K., H.M., C.B. and K.B. collected data; L.K., R.J.H., T.H., J.D., A.K., H.M., C.B. P.Z., O.H. and K.B analyzed and interpreted data; L.K., M.D., G.M., W.H. and K.B. cared for the patients; L.K., R.J.H., O.H. and K.B. wrote the manuscript. All authors approved the final version of the manuscript.

## Supporting information


**Table S1.** Genes included in the Hematology panel design divided by disease group.
**Table S2.** Pertinent data of the 11 patients with anemia.
**Table S3.** Pertinent data of the two patients with thrombocytopenia.
**Table S4.** Pertinent data of the patient with leukopenia.
**Table S5.** Pertinent data of the patient with bicytopenia.
**Table S6.** Pertinent data of the two patients with pancytopenia.
**Figure S1.** MLPA results with the FANCA kits P031‐B2 (left) and P032‐B2 (right). Results from normal controls (peak ratio 1 corresponding to 2 copies) and the twin girls harboring a biallelic deletion of exons 6‐31 of the *FANCA* gene (peak ratio 0) are shown.
