# Supplementary material for: Targeted mutation screening of 292 candidate genes in 38 children with inborn haematological cytopenias efficiently identifies novel disease‐causing mutations
Source: Br J Haematol. 2018 May 24;182(2):251–8. doi: 10.1111/bjh.15389 (PMC6079646; doi:10.1111/bjh.15389)
Supplement: Supplementary file 1 — Table S1. Genes included in the Hematology panel design divided by disease group. Table S2. Pertinent data of the 11 patients with anemia. Table S3. Pertinent data of the two patients with thrombocytopenia. Table S4. Pertinent data of the patient with leukopenia. Table S5. Pertinent data of the patient with bicytopenia. Table S6. Pertinent data of the two patients with pancytopenia. Figure S1. MLPA results with the FANCA kits P031‐B2 (left) and P032‐B2 (right). Results from normal controls (peak ratio 1 corresponding to 2 copies) and the twin girls harboring a biallelic deletion of exons 6‐31 of the FANCA gene (peak ratio 0) are shown. [file BJH-182-251-s001.doc]

**Supporting information to**

**Targeted mutation screening of 292 candidate genes in 38 children with inborn haematological cytopenias efficiently identifies novel disease-causing mutations**

Leo Kager, Raúl Jimenez Heredia, Tatjana Hirschmugl, Ana Krolo, Heiko Müller, Christoph Bock, Petra Zeitlhofer, Michael Dworzak, Georg Mann, Wolfgang Holter, Oskar Haas and Kaan Boztug

**Supplemental Results**

We here established a comprehensive NGS-based panel covering 292 candidate genes for different types of inborn hematologic diseases (Supplementary Table 1). After variant prioritization, a total of 17/38 (44.7%) patients remained in whom potential disease-causing mutations were identified. In 21/38 (55.3%) patients our approach did not reveal disease-causing mutations, and a sub-fraction of these patients might be interesting for further genetic investigation, e.g. by exome or whole-genome sequencing.

In the sub-cohort of 17 patients, the median number of identified potentially disease-causing genes per patient was two (range, one to 7, see below); and table was created for each of the 17 patients, which contain details on the most likely disease-causing variant(s), as per the following format:

| **GENE^1^** | **CHR^2^** | **POS^3^** | **SNP_ID^4^** | **REF^5^** | **ALT^6^** | **EFFECT^7^** | **AA^8^** | **TRANSCRIPT^9^** | **ExAC MAF^10^** | **READS^11^** | **PolyPhen^12^** | **SIFT^13^** | **CADD^14^** |
| --- | --- | --- | --- | --- | --- | --- | --- | --- | --- | --- | --- | --- | --- |

^1^the identified gene(s) symbol (GENE) according to the HUGO Gene Nomenclature Committee (<https://www.genenames.org/>),

^2^the chromosome (CHR) on which the gene is localized (<https://www.ncbi.nlm.nih.gov/gene/>),

^3^the position (POS) of the identified variant(s) in the GRCh37/hg19 assembly (<https://www.ncbi.nlm.nih.gov/assembly/GCF_000001405.13/>),

^4^the primary identifier for variants (SNP_ID) in the Single Nucleotide Polymorphism Database (dbSNP) of Nucleotide Sequence Variation (<https://www.ncbi.nlm.nih.gov/books/NBK21088/>),

^5^the nucleotide present in the reference (REF) genome GRCh37/hg19,

^6^the nucleotide present in the patient alternatively (ALT) to the reference,

^7^the effect (EFFECT) which is caused by the variant given by the *snpeff* annotation in the transcript (<http://snpeff.sourceforge.net/>),

^8^the amino acid change (AA) annotated following the guidelines from the Human Genome Variation Society (<http://varnomen.hgvs.org/>),

^9^the transcript (TRANSCRIPT) for which the gDNA and protein change is described,

^10^the minor allele frequency (ExAC MAF) provided for each specific variant in all populations/ethnicities in the Exome Aggregation Consortium (<http://exac.broadinstitute.org/>),

^11^the number of reads (READS) covering the specific position, and the results from in silico analyses using the prediction tools:

^12^Prediction program: PolyPhen (<http://genetics.bwh.harvard.edu/pph2/>) (Adzhub*ei, et* al 2010),

^13^Prediction program: SIFT (<http://sift.jcvi.org/>) (Ng and Henikoff 2003),

^14^Prediction program: CADD (<http://cadd.gs.washington.edu/>) (Kirch*er, et* al 2014).

In the tables, the identified disease-causing genes and their variants are depicted in **bold and are underlined**. Variants which have been reported to be disease-causing (i.e., causing the hematological phenotype cytopenia) in heterozygous individuals are depicted in **bold**. Finally, the identified disease-causing variants were compared with data from the scientific literature (<https://www.ncbi.nlm.nih.gov/pubmed/>, search terms: identified variant(s), respective clinical phenotype(s)); and the results for each patient are provided below.

Overall, we identified homozygous disease-causing variants in 3/17 patients (patients 6, 16 and 17; homozygous variants in *PKLR*, *FANCA* and *NHEJ1*, respectively), compound heterozygous disease-causing variants in 2/17 (patients 9 and 10; compound heterozygous variants in *CDAN1*) and heterozygous disease-causing variants in the remaining 12/17 patients (patients 1 and 2 with heterozygous variants in *SPTB*, patients 3, 4 and 5 with heterozygous variants in *ANK1*, patients 7 and 8 with heterozygous variants in *PIEZO1*, patient 11 with a heterozygous variant in *RPS29*, patient 12 with a heterozygous variant in *RUNX1*, patient 13 with a heterozygous variant in *MYH9*, patient 14 with a heterozygous variant in *GATA2* and patient 15 with a heterozygous variant in *RPL5*). More details are presented below.

**Patient 1**

**Homozygous Variants:** No homozygous variants left

**Heterozygous Variants:** see Table

| **GENE** | **CHR** | **POS** | **SNP_ID** | **REF** | **ALT** | **EFFECT** | **AA** | **TRANSCRIPT** | **ExAC MAF** | **READS** | **PolyPhen** | **SIFT** | **CADD** |
| --- | --- | --- | --- | --- | --- | --- | --- | --- | --- | --- | --- | --- | --- |
| ***SPTB*** | **14** | **65235810** | **.** | **C** | **G** | **FRAME SHIFT** | **M1988Sfs*7** | **ENST00000389722** | **NA** | **197** | **benign** | **tolerated** | **11,06** |
| ***SPTB*** | **14** | **65235811** | **.** | **AT** | **A** |  |  | **ENST00000389722** | **NA** | **197** | **NA** | **NA** | **23,1** |
| *VPS13B* | 8 | 100833627 | . | G | A | MISSENSE | G3059S | ENST00000358544 | < 0.01 | 236 | possibly damaging | tolerated | 23,7 |

**Abbreviations.** NA, not annotated; SPTB, spectrin, beta, erythrocytic; VPS13B, vacuolar protein sorting 13 homolog B.

**Interpretation:** The patient had HS phenotype and HS diagnosis was confirmed via Eosin-5-maleimide binding testing (EMA, Supplemental Table 2). Both detected *SPTB* deletion/insertion variants were found on the same allele creating a frameshift mutation and a premature stop codon after amino acid 1993, resulting in an erroneous truncated protein. The healthy mother of the patient carries wild-type *SPTB* alleles and the father was not available for analysis (deceased), but was reported to have had no evidence for HS. Pathogenic *SPTB* variants account for 20% of hereditary spherocytosis (HS) patients (An and Mohandas 2008); and the identified variants are considered to be the *de novo* Mendelian genotype that causes the phenotype HS in patient 1.

Only homozygous *VPS13B* disease-causing variants were reported to cause Cohen syndrome. The patient, however, does not have a Cohen syndrome phenotype and carries a heterozygote variant only.

**Patient 2**

**Homozygous Variants:** No homozygous variants left

**Heterozygous Variants:** see Table

| **GENE** | **CHR** | **POS** | **SNP_ID** | **REF** | **ALT** | **EFFECT** | **AA** | **TRANSCRIPT** | **ExAC MAF** | **READS** | **PolyPhen** | **SIFT** | **CADD** |
| --- | --- | --- | --- | --- | --- | --- | --- | --- | --- | --- | --- | --- | --- |
| ***SPTB*** | **14** | **65246606** | **.** | **T** | **TC** | **FRAME SHIFT** | **E1437Gfs*54** | **ENST00000389722** | **NA** | **732** | **NA** | **NA** | **35** |

**Abbreviations.** NA, not annotated; SPTB, spectrin, beta, erythrocytic

**Interpretation:** The patient had HS phenotype and HS diagnosis was confirmed via EMA testing (Supplemental Table 2). The heterozygous *SPTB* insertion/duplication variant causes a frameshift mutation and a premature stop codon after amino acid 1489, resulting in an erroneous truncated protein. Moreover, there is a very high CADD prediction score (i.e., 35) for the identified variant. The father has the same phenotype (HS) and carries the same heterozygous *SPTB* insertion/duplication variant as the female patient. The identified variant is considered to be the autosomal dominant inherited Mendelian genotype that causes the phenotype HS in patient 2.

**Patient 3**

**Homozygous Variants:** No homozygous variants left

**Heterozygous Variants:** see Table

| **GENE** | **CHR** | **POS** | **SNP_ID** | **REF** | **ALT** | **EFFECT** | **AA** | **TRANSCRIPT** | **ExAC MAF** | **READS** | **PolyPhen** | **SIFT** | **CADD** |
| --- | --- | --- | --- | --- | --- | --- | --- | --- | --- | --- | --- | --- | --- |
| ***ANK1*** | **8** | **41584836** | **.** | **G** | **A** | **STOP GAIN** | **Q153*** | **ENST00000396942** | **NA** | **248** | **NA** | **NA** | **38** |
| *FANCG* | 9 | 35076478 | . | G | C | MISSENSE | Q343E | ENST00000378643 | NA | 233 | benign | tolerated | 13,04 |

**Abbreviations.** ANK1, ankyrin 1, FANCG, Fanconi anemia complementation group G, NA, not annotated

**Interpretation:** The patient had HS phenotype and HS diagnosis was confirmed via glycerol lysis and kryohemolysis testing (Supplemental Table 2). The heterozygous missense variant in *ANK1* causes a premature stop codon in amino acid 153, resulting in an erroneous truncated protein. Moreover, there is a very high CADD prediction score (i.e., 38) for the identified variant. The parents were not analyzed yet, but the father has the same phenotype (HS). Analysis of parents is planned. Pathogenic *ANK1* variants account for 50% of hereditary spherocytosis (HS) patients (An and Mohandas 2008); and the identified variant is considered to the autosomal dominant inherited Mendelian genotype that causes the phenotype HS in patient 3.

Only homozygous *FANCG* disease-causing variants were reported in patients with Fanconi anemia; and FA is not the phenotype identified in patient 3. Moreover, the identified variant in *FANCG* has low disease-causing prediction scores, and the patient carries a heterozygote variant only.

**Patient 4**

**Homozygous Variants:** No homozygous variants left

**Heterozygous Variants: see Table**

| GENE | CHR | POS | SNP_ID | REF | ALT | EFFECT | AA | TRANSCRIPT | ExAC MAF | READS | PolyPhen | SIFT | CADD |
| --- | --- | --- | --- | --- | --- | --- | --- | --- | --- | --- | --- | --- | --- |
| ***ANK1*** | **8** | **41543669** | **.** | **CTGTT** | **C** | **FRAME SHIFT** | **N1504Wfs*17** | **ENST00000396942** | **NA** | **621** | **NA** | **NA** | **35** |
| ***ATR*** | **3** | **1,42E+08** | **rs201492267** | **A** | **C** | **MISSENSE** | **S1616A** | **ENST00000350721** | **<0.01** | **96** | **benign** | **tolerated** | **0,045** |
| ***CARD11*** | **7** | **2954961** | **rs375594527** | **C** | **T** | **MISSENSE** | **E917K** | **ENST00000396946** | **<0.01** | **224** | **benign** | **tolerated** | **24** |
| *WFS1* | 4 | 6303900 | . | G | A | MISSENSE | R793H | ENST00000226760 | <0.01 | 1484 | benign | tolerated | 2,624 |
| *VPS13B* | 8 | 1E+08 | rs372625091 | A | G | MISSENSE | T543A | ENST00000358544 | <0.01 | 210 | possibly damaging | tolerated | 23,3 |

**Abbreviations.** ANK1, ankyrin 1; ATR, ATR serine/threonine kinase; CARD11, caspase recruitment domain family member 11; NA, not annotated; VPS13B, vacuolar protein sorting 13 homolog B; WFS1, wolframin ER transmembrane glycoprotein.

**Interpretation:** The patient had HS phenotype and HS diagnosis was confirmed via EMA testing (Supplemental Table 2). The heterozygous deletion variant in *ANK1* causes a frameshift mutation and a premature stop codon after amino acid 1519, resulting in an erroneous truncated protein. Moreover, there is a high CADD prediction score (i.e., 35) for the identified variant. The father has the same phenotype (HS) and carries the same heterozygous deletion variant. The healthy mother carries *ANK1* wild-type alleles. The identified *ANK1* variant is considered to be the autosomal dominant inherited Mendelian genotype that causes the phenotype HS in patient 4 and his father.

The variants in *ATR* (pathogenic variants cause Seckel Syndrome) and *CARD11* (pathogenic variants cause autoimmune lymphoproliferative syndrome) do not fit the phenotype and do have low prediction scores.

Only homozygous variants in *VPS13B* and *WFS1* are known to be disease-causing (Cohen syndrome and Wolfram syndrome 1, respectively). Patient 4 has no phenotypic features of Cohen or Wolfram syndromes and carries heterozygous variants only.

**Patient 5**

**Homozygous Variants:** No homozygous variants left

**Heterozygous Variants: see Table**

| **GENE** | **CHR** | **POS** | **SNP_ID** | **REF** | **ALT** | **EFFECT** | **AA** | **TRANSCRIPT** | **ExAC MAF** | **READS** | **PolyPhen** | **SIFT** | **CADD** |
| --- | --- | --- | --- | --- | --- | --- | --- | --- | --- | --- | --- | --- | --- |
| ***ANK1*** | **8** | **41571688** | **.** | **GCGGGGAGCCGCCC** | **G** | **FRAME SHIFT** | **p.G625Tfs*41** | **ENST00000396942** | **NA** | **496** | **NA** | **NA** | **36** |
| *ABCA1* | 9 | 107550310 | . | A | T | MISSENSE | L2032Q | ENST00000374736 | NA | 330 | probably damaging | deleterious | 32 |

**Abbreviations.** ABCA1, ATP binding cassette subfamily A member 1; ANK1, ankyrin 1; NA, not annotated

**Interpretation:** The patient had HS phenotype and HS diagnosis was confirmed via EMA testing (Supplemental Table 2). The heterozygous deletion variant in *ANK1* causes a frameshift mutation and a premature stop codon after amino acid 664, resulting in an erroneous truncated protein. Moreover, there is a very high CADD prediction score (i.e., 36) for the identified variant. The father and her sister have the same phenotype (HS) and carry the same heterozygous deletion variant. The healthy mother carries *ANK1* wild-type alleles. The identified *ANK1* variant is considered to be the autosomal dominant inherited Mendelian genotype that causes the phenotype HS in patient 5, her father and her sister.

Homozygous damaging variants in *ABCA1* have been identified to cause Tangier disease. No abnormalities in lipid metabolism, however, have been observed in patient 5; and she carries a heterozygous variant in *ABCA1* only.

**Patient 6**

**Homozygous Variants:** see Table

| **GENE** | **CHR** | **POS** | **SNP_ID** | **REF** | **ALT** | **EFFECT** | **AA** | **TRANSCRIPT** | **ExAC MAF** | **READS** | **PolyPhen** | **SIFT** | **CADD** |
| --- | --- | --- | --- | --- | --- | --- | --- | --- | --- | --- | --- | --- | --- |
| ***PKLR*** | **1** | **155260413** | **.** | **G** | **C** | **MISSENSE** | **R559G** | **ENST00000342741** | **NA** | **16** | **benign** | **deleterious** | **24,3** |

**Heterozygous Variants: see Table**

| **GENE** | **CHR** | **POS** | **SNP_ID** | **REF** | **ALT** | **EFFECT** | **AA** | **TRANSCRIPT** | **ExAC MAF** | **READS** | **PolyPhen** | **SIFT** | **CADD** |
| --- | --- | --- | --- | --- | --- | --- | --- | --- | --- | --- | --- | --- | --- |
| *TCN2* | 22 | 31019016 | rs367605153 | G | A | MISSENSE | G390R | ENST00000215838 | <0.01 | 387 | benign | tolerated | 13,09 |
| *CUBN* | 10 | 16994388 | . | G | T | MISSENSE | A1619D | ENST00000377833 | NA | 106 | benign | deleterious | 16,75 |
| *SLX4* | 16 | 3632408 | . | G | A | MISSENSE | R1814C | ENST00000294008 | <0.01 | 1045 | probably damaging | deleterious | 26,7 |

**Abbreviations.** CUBN, cubilin; NA, not annotated; PKLR, pyruvate kinase L/R; SLX4, SLX4 structure-specific endonuclease subunit; TCN2, transcobalamin 2.

**Interpretation:** The patient’s phenotype was moderate macrocytic chronic hemolytic anemia of unknown cause. The homozygous missense variant in *PKLR*, which affects the C-domain of the PKLR protein, was already identified to be disease-causing (Baroncia*ni, et* al 1995, Unal and Gumruk 2015). In-line with these findings, this variant has a moderate CADD score (i.e., 24.3) and is predicted to be deleterious via SIFT. The consanguine, healthy parents are heterozygous carriers of the disease-causing *PKLR* variant, but have normal pyruvate kinase (PK) enzyme activities. Patient 6 had a PK enzyme activity in the lower normal range 11 IE/gHb (normal range, 10 to 20 IE/gHb). However, at the time of analysis there was a reticulocytosis (18.4%), and other red blood cell enzyme activities were markedly elevated; e.g. glucose-6-phosphate dehydrogenase 17.8 IE/gHb (normal 9 – 14 IE/gHb). Therefore, it can be concluded, that the patient has PK deficiency caused by the autosomal recessive inherited homozygous missense variant in *PKLR*.

Homozygous damaging variants in *TCN2, SLX4* and *CUBN*, have been reported to cause transcobalamin deficiency, Fanconi anemia and Imerslund Gresbeck Syndrome, respectively. No abnormalities in folate, vitamin B12, homocysteine metabolism and diepoxybutane (DEB) testing, however, have been observed in patient 6. Moreover, she carries heterozygous variants in *TCN2, SLX4* and *CUBN* only.

**Patient 7**

**Homozygous Variants:** No homozygous variants left

**Heterozygous Variants: see Table**

| GENE | CHR | POS | SNP_ID | REF | ALT | EFFECT | AA | TRANSCRIPT | ExAC MAF | READS | PolyPhen | SIFT | CADD |
| --- | --- | --- | --- | --- | --- | --- | --- | --- | --- | --- | --- | --- | --- |
| ***PIEZO1*** | **16** | **88782212** | **.** | **C** | **T** | **MISSENSE** | **R2456H** | **ENST00000301015** | **NA** | **109** | **probably damaging** | **deleterious** | **27,5** |
| ***CD46*** | **1** | **2,08E+08** | **.** | **CAG** | **C** | **FRAME SHIFT** | **Q238Hfs*17** | **ENST00000322875** | **NA** | **146** | **NA** | **NA** | **23,8** |
| *USB1* | 16 | 58045017 | . | G | A | MISSENSE | G182E | ENST00000423271 | NA | 619 | NA | NA | 1.824 |

**Abbreviations.** CD46, CD46 molecule; PIEZO1, piezo type mechanosensitive ion channel component 1; USB1, U6 SnRNA Biogenesis Phosphodiesterase 1; NA, not annotated.

**Interpretation:** The patient’s phenotype was mild chronic macrocytic hemolytic anemia with high hyperbilirubinemia of unknown cause. The heterozygous missense variant R2456H in *PIEZO1* affects a highly conserved region and has already been identified as a cause of the very rare disease hereditary xerocytosis (HX) (Zarychans*ki, et* al 2012). In-line with these previous results, this variant has a high CADD score (i.e., 27.5) and is predicted to be deleterious via SIFT and probably damaging via PolyPhen. R2456H is the HX phenotype-causing variant in patient 7. The family members have not been analyzed yet, but the mother, her father and her grandfather have also been reported to suffer from ‘familial hyperbilirubinemia’. Genetic analyses of family members are planned.

Heterozygous damaging variants in *CD46* can cause atypical haemolytic uremic syndrome (aHUS) (Liszewski and Atkinson 2015). The patient doses not have the phenotype of aHUS; therefore this observed variant is unlikely to cause aHUS or to contribute to the phenotype chronic, macrocytic, hemolytic anemia. Homozygous variants in *USB1* have been reported to cause poikiloderma with neutropenia; and the patient does not have this phenotype and he carries a heterozygous variant in *USB1* (with a very low CADD score) only.

**Patient 8**

**Homozygous Variants:** No homozygous variants left

**Heterozygous Variants: see Table**

| GENE | CHR | POS | SNP_ID | REF | ALT | EFFECT | AA | TRANSCRIPT | ExAC MAF | READS | PolyPhen | SIFT | CADD |
| --- | --- | --- | --- | --- | --- | --- | --- | --- | --- | --- | --- | --- | --- |
| ***PIEZO1*** | **16** | **88791904** | **.** | **T** | **C** | **MISSENSE** | **Q1361R** | **ENST00000301015** | **NA** | **1012** | **probably damaging** | **deleterious** | **27,1** |
| ***BRCA1*** | **17** | **41243919** | **.** | **T** | **C** | **MISSENSE** | **E1210G** | **ENST00000471181** | **NA** | **195** | **benign** | **deleterious** | **23,5** |
| ***TBX1*** | **22** | **19754004** | **.** | **G** | **C** | **MISSENSE** | **V368L** | **ENST00000332710** | **NA** | **217** | **benign** | **tolerated** | **20,6** |
| ***PIEZO1*** | **16** | **88787595** | **rs200031013** | **G** | **A** | **MISSENSE** | **R1883W** | **ENST00000301015** | **< 0.01** | **418** | **benign** | **deleterious** | **23** |
| ***SPTB*** | **14** | **65268950** | **rs184200762** | **G** | **A** | **MISSENSE** | **T187M** | **ENST00000389722** | **< 0.01** | **345** | **probably damaging** | **deleterious** | **33** |
| ***TINF2*** | **14** | **24709123** | **.** | **GT** | **G** | **FRAME SHIFT** | **N412Tfs10*** | **ENST00000267415** | **NA** | **506** | **NA** | **NA** | **33** |
| *FANCA* | 16 | 89818581 | rs142377616 | G | A | MISSENSE | R1011C | ENST00000389301 | < 0.01 | 529 | possibly damaging | tolerated | 22,7 |
| *PRKDC* | 8 | 48694982 | . | C | A | MISSENSE | A3784S | ENST00000314191 | NA | 459 | benign | tolerated | 9,307 |

**Abbreviations.** BRCA1, DNA repair associated; FANCA, Fanconi anemia complementation group A; NA, not annotated; PIEZO1, piezo type mechanosensitive ion channel component 1; PRKDC, protein kinase, DNA-activated, catalytic polypeptide; SPTB, spectrin, beta, erythrocytic; TBX1, T-box 1; TINF2, TERF1 interacting nuclear factor 2;.

**Interpretation:** The patient’s phenotype was mild chronic macrocytic hemolytic anemia with high hyperbilirubinemia of unknown cause. The identified heterozygous missense variant in *PIEZO1*, i.e., Q1361R, is localized close to an already identified HX-causing variant, i.e., R1358P (Albuiss*on, et* al 2013). Moreover, this variant has a high CADD score (i.e., 27.1) and is predicted to be deleterious via SIFT and probably damaging via PolyPhen. The healthy consanguine parents do not carry the R1358P *PIEZO1* variant; and this de novo variant is considered to cause the HX phenotype in patient 8.

In addition, the patient and her mother carry a heterozygous variant (i.e., R1883W) in *PIEZO1*. This variant has a lower CADD score (i.e., 23 versus 27.1) and is predicted to be benign in the PolyPhen analysis. As the mother is healthy, this variant unlikely contributes to the HX phenotype in patient 8.

Of note, a novel heterozygous missense mutation, i.e., T187M, in SPTB was identified in the patient and in her healthy father. This variant has a high CADD score (i.e., 33) and is predicted to be deleterious via SIFT and probably damaging via PolyPhen. However, as neither the father nor the patient have evidence for hereditary spherocytosis, this variant cannot be considered as a disease-causing variant.

Other identified heterozygous variants that may cause diseases were identified in *TINF2* (dyskeratosis congenita (DC), CADD score 33), *BRCA1* (Fanconi anemia, CADD score 23.5) and *TBX1* (DiGeorge syndrome, CADD score 20.6). The patient, however, does not have a phenotype of these diseases.

Homozygous damaging variants in *PRKDC* and in *FANCA* have been reported to cause (severe) combined immunodeficiency ((S)CID) and Fanconi anemia, respectively. Patient 8 does not have these phenotypes and she carries heterozygous variants in *PRKDC and FANCA* only.

**Patient 9**

**Homozygous Variants:** No homozygous variants left

**Heterozygous Variants: see Table**

| GENE | CR | POS | SNP_ID | REF | ALT | EFFECT | AA | TRANSCRIPT | ExAC MAF | READS | Poly  Phen | SIFT | CADD |
| --- | --- | --- | --- | --- | --- | --- | --- | --- | --- | --- | --- | --- | --- |
| ***CDAN1*** | **15** | **43022926** | **rs375339408** | **G** | **A** | **STOP GAINED** | **R682*** | **ENST00000356231** | **NA** | **40** | **NA** | **NA** | **38** |
| ***CDAN1*** | **15** | **43016798** | **.** | **A** | **G** | **MISSENSE** | **L1192S** | **ENST00000356231** | **NA** | **88** | **probably damaging** | **deleterious** | **27,3** |
| ***GP1BA*** | **17** | **4837171** | **.** | **GGAGCCCACCTCAGAGCCCGCCCCCAGCCCGACCACCCCA** | **G** | **CODON DELE-TION** | **EPTSEPAPSPTTP425** | **ENST00000329125** | **NA** | **52** | **NA** | **NA** | **6,824** |
| ***APOB*** | **2** | **21245889** | **rs12714097** | **G** | **A** | **MISSENSE** | **P877L** | **ENST00000233242** | **NA** | **362** | **probably damaging** | **deleterious** | **29,1** |
| *CEP152* | 15 | 49048132 | rs74553953 | G | C | MISSENSE | L1105V | ENST00000380950 | < 0.01 | 738 | benign | tolerated | 0.014 |
| *ITGB2* | 21 | 46311778 | rs138659490 | C | T | MISSENSE | S453N | ENST00000302347 | < 0.01 | 1098 | benign | tolerated | 1.922 |
| *GSS* | 20 | 33517271 | . | G | A | MISSENSE | R412W | ENST00000216951 | NA | 662 | probably damaging | deleterious | 34 |

**Abbreviations.** APOB, Apolipoprotein B; CDAN1, codanin 1; CEP152, centrosomal protein 152; GP1BA, glycoprotein Ib platelet alpha subunit; GSS, glutathione synthetase; ITGB2, integrin subunit beta 2; NA, not annotated.

**Interpretation:** The patient’s phenotype was moderate (transfusion-dependent in infancy), chronic, macrocytic, hemolytic anemia of unknown cause (bone marrow investigation was initially refused by parents – consent was given after identification of *CDAN1* variants). We identified compound heterozygous damaging variants in *CDAN1*, which can cause the phenotype congenital dyserythropoietic anemia type 1 (CDA1) (Dga*ny, et* al 2002). The p.R682X nonsense variant, which had been identified as CDA1-causing variant by Dgany et al. (Dga*ny, et* al 2002), was inherited from the healthy mother. This variant has a very high CADD score (i.e., 38). The novel disease-causing missense *CDAN1* variant p.L1192S in exon 28 was inherited by the healthy father and has also a high CADD score (i.e., 29.1) and is predicted to be probably damaging via PolyPhen and deleterious via SIFT. The phenotype CDA1 in patient 9 is caused by the autosomal recessive inherited *CDAN1* variants p.R682X and p.L1192S.

Other identified heterozygous variants that may cause diseases were identified in *APOB* (homozygous familial hypobetalipoproteinemia, CADD score 29.1), and *GP1BA* (Bernard-Soulier syndrome (BSS), low CADD score of 6.8). Homozygous damaging variants in *APOB* can cause acanthocytosis. However, the patient has a heterozygous variant only and no typical alteration in blood lipid metabolism. In addition, the patient has no BSS phenotype.

Homozygous damaging variants in *CEP152, ITGB2* and *GSS* have been reported to cause Seckel syndrome (SS), leukocyte adhesion deficiency (LAD) 2 and glutathione synthetase deficiency, respectively. Patient 9 does not have these phenotypes and he carries heterozygous variants in these genes only.

**Patient 10**

**Homozygous Variants:** No homozygous variants left

**Heterozygous Variants:** see Table

| **GENE** | **CHR** | **POS** | **SNP_ID** | **REF** | **ALT** | **EFFECT** | **AA** | **TRANSCRIPT** | **ExAC MAF** | **READS** | **PolyPhen** | **SIFT** | **CADD** |
| --- | --- | --- | --- | --- | --- | --- | --- | --- | --- | --- | --- | --- | --- |
| ***CDAN1*** | **15** | **43022955** | **rs120074167** | **G** | **A** | **MISSENSE** | **P672L** | **ENST00000356231** | **<0.01** | **423** | **probably damaging** | **tolerated** | **25,8** |
| ***CDAN1*** | **15** | **43026492** | **.** | **G** | **A** | **MISSENSE** | **R397W** | **ENST00000356231** | **<0.01** | **417** | **probably damaging** | **deleterious** | **34** |
| *STAT5B* | 17 | 40376874 | rs199894785 | G | A | MISSENSE | R100C | ENST00000293328 | <0.01 | 393 | benign | deleterious | 29 |

**Abbreviations.** CDAN1, codanin 1; NA, not annotated; STAT5B, signal transducer and activator of transcription 5B.

**Interpretation:** The patient’s phenotype was moderate (transfusion-dependent in infancy), chronic, macrocytic, hemolytic anemia associated with cardiac, skeletal, and Arnold Chiari type 1 malformations of unknown cause. We identified compound heterozygous damaging variants in *CDAN1*. The p.P672L (sometimes also designated as p.P671L) missense variant, which had been identified as CDA1-causing variant earlier (Dga*ny, et* al 2002), was inherited from the healthy father. This variant has a CADD score of 25.8. The novel disease-causing missense *CDAN1* variant p.R397W in exon 7 was inherited by the healthy mother, has a very high CADD score of 34 and is predicted to be probably damaging in Polyphen and deleterious in SIFT. The phenotype CDA1 in patient 10 is caused by the autosomal recessive inherited *CDAN1* variants p.P672L and p.R397W.

Homozygous damaging variants in *STAT5B* have been identified to cause immune dysregulation, polyendocrinopathy, enteropathy, X-linked (IPEX)-like. Patient 10 does not have these phenotypes and he carries a heterozygous variant only.

**Patient 11**

**Homozygous Variants:** No homozygous variants left

**Heterozygous Variants:** see Table

| **GENE** | **CHR** | **POS** | **SNP_ID** | **REF** | **ALT** | **EFFECT** | **AA** | **TRANSCRIPT** | **ExAC MAF** | **READS** | **Polyphen** | **SIFT** | **CADD** |
| --- | --- | --- | --- | --- | --- | --- | --- | --- | --- | --- | --- | --- | --- |
| ***RPS29*** | **14** | **50052691** | **.** | **C** | **T** | **MISSENSE** | **A47T** | **ENST00000396020** | **NA** | **392** | **probably damaging** | **deleterious** | **35** |
| ***PIEZO1*** | **16** | **88788078** | **.** | **G** | **C** | **MISSENSE** | **H1757Q** | **ENST00000301015** | **NA** | **454** | **benign** | **tolerated** | **14.00** |

**Abbreviations.** NA, not annotated; PIEZO1, piezo type mechanosensitive ion channel component 1; RPS29, ribosomal protein S29.

**Interpretation:** The patient’s phenotype was Diamond-Blackfan anemia (DBA); and we identified a novel heterozygous disease-causing variant in *RPS29*. This p.A47T missense variant resides in a highly conserved region within exon 2 of *RPS29*; and two families with multiple affected individuals have been reported to carry heterozygous disease-causing variants (i.e., p.I31F and p.I50T) within this region in *RPS29* (Mirabel*lo, et* al 2014). The p.A47T variant has a very high CADD score (i.e., 35) and is predicted to be probably damaging (PolyPhen) and deleterious (SIFT). The novel disease-causing missense variant was inherited autosomal dominant by the healthy father; the mother carries two wild-type alleles.

One other heterozygous variant, that may cause diseases was identified in *PIEZO1* (HX, CADD score 14). However, the patient has no HX phenotype and this variant is predicted to be benign (PolyPhen) and tolerated (SIFT).

**Patient 12**

**Homozygous Variants:** No homozygous variants left

**Heterozygous Variants:** see Table

| **GENE** | **CHR** | **POS** | **SNP_ID** | **REF** | **ALT** | **EFFECT** | **AA** | **TRANSCRIPT** | **ExAC MAF** | **READS** | **PolyPhen** | **SIFT** | **CADD** |
| --- | --- | --- | --- | --- | --- | --- | --- | --- | --- | --- | --- | --- | --- |
| ***RUNX1*** | **21** | **36252869** | **.** | **C** | **G** | **MISSENSE** | **G165R** | **ENST00000300305** | **NA** | **145** | **probably damaging** | **deleterious** | **34** |

**Abbreviations.** NA, not annotated; RUNX1, runt related transcription factor 1.

**Interpretation:** The patient’s phenotype was inborn, mild, normocytic thrombocytopenia of unknown cause. We identified a novel heterozygous, predicted disease-causing variant in *RUNX1.* This p.G165R missense variant resides in the highly conserved Runt homology domain within exon 5 of *RUNX1*; and a nonsense variant (i.e., p.R166X) adjacent to the herein identified variant was recently described in a 39-years old male with mild normocytic thrombocytopenia (77 G/l) and leukopenia (3.2 G/l) (Kanagal-Shaman*na, et* al 2017). The p.G165R variant has a very high CADD score (i.e., 34) and is predicted to be probably damaging (Polyphen) and deleterious (SIFT). No other family members were found to have thrombocytopenia; and genetic analyses of all family members are planned. Moreover, functional testing of the patient’s thrombocytes is planned.

**Patient 13**

**Homozygous Variants:** No homozygous variants left

**Heterozygous Variants:** see Table

| **GENE** | **CHR** | **POS** | **SNP_ID** | **REF** | **ALT** | **EFFECT** | **AA** | **TRANSCRIPT** | **ExAC MAF** | **READS** | **PolyPhen** | **SIFT** | **CADD** |
| --- | --- | --- | --- | --- | --- | --- | --- | --- | --- | --- | --- | --- | --- |
| ***MYH9*** | **22** | **36744995** | **rs121913657** | **G** | **A** | **MISSENSE** | **S96L** | **ENST00000216181** | **NA** | **272** | **probably damaging** | **deleterious** | **32** |
| *BRCA2* | 13 | 32910812 | rs55968715 | A | G | MISSENSE | T774A | ENST00000380152 | <0.01 | 651 | benign | tolerated | 0,001 |

**Abbreviations.** BRCA2, BRCA2, DNA repair associated; MYH9, myosin heavy chain 9, NA, not annotated.

**Interpretation:** The patient’s phenotype was inborn, severe, macrocytic (giant platelets) thrombocytopenia of unknown cause. We identified a known heterozygous, disease-causing variant in *MYH9.* This p.S96L missense variant resides in the head domain (which interacts with actin and binds ATP) within exon 2 of *MYH9*; and variants in this region are known to cause a phenotype with very large platelets and low thrombocyte counts (Nor*is, et* al 2014, Pec*ci, et* al 2014). The p.S96L variant has a very high CADD score (i.e., 32) and is predicted to be probably damaging (Polyphen) and deleterious (SIFT). No other family members were found to have thrombocytopenia and have *MYH9* wild-type alleles.

Homozygous damaging variants in *BRCA2* can cause FA. The patient does not have a FA phenotype and carries a heterozygous variant. Moreover, the identified variant has an extremely low CADD score (i.e., 0.001) and is predicted to be benign (PolyPhen) and tolerated (SIFT).

**Patient 14**

**Homozygous Variants:** No homozygous variants left

**Heterozygous Variants:** see Table

| **GENE** | **CHR** | **POS** | **SNP_ID** | **REF** | **ALT** | **EFFECT** | **AA** | **TRANSCRIPT** | **ExAC MAF** | **READS** | **PolyPhen** | **SIFT** | **CADD** |
| --- | --- | --- | --- | --- | --- | --- | --- | --- | --- | --- | --- | --- | --- |
| ***GATA2*** | **3** | **128205754** | **rs143590990** | **G** | **C** | **MISSENSE** | **P41A** | **rs143590990** | **< 0.01** | **418** | **probably damaging** | **tolerated** | **23** |
| *SH2D1A* | X | 123480610 | rs199639961 | G | A | MISSENSE | V40M | rs199639961 | < 0.01 | 198 | benign | tolerated | 22,9 |
| *THBD* | 20 | 23029958 | . | C | T | MISSENSE | V62M | . | < 0.01 | 189 | probably damaging | deleterious | 29,6 |

**Abbreviations.** GATA2, GATA binding protein 2; SH2D1A, SH2 domain containing 1A; THBD, thrombomodulin.

**Interpretation:** The patient’s phenotype was chronic moderate leukopenia with recurrent fever and aphthous oral lesions. We identified a very rare, but already known heterozygous, disease-causing variant in *GATA2.* This p.P41A missense variant in exon 4 of *GATA2* was previously identified in a 48 years-old with myelodysplastic syndrome (MDS) in a family with multiple cancer cases (3 family members were reported to have MDS or chronic myeloid leukemia (CML)) (Hol*me, et* al 2012). Our female patient did not have any evidence for MDS, but within her family, cancer cases including MDS were reported. The living family members were not analyzed for *GATA2* variants yet. The p.P41A missense variant has a moderate CADD score (i.e., 23) and is predicted to be probably damaging by Polyphen, but tolerated via SIFT.

Disease-causing variants in the *SH2D1A* gene, which resides on chromosome X, cause X-linked lymphoproliferative disease (LPD) in males. In very rare cases, heterozygous females may be symptomatic due to skewed X-chromosome inactivation. Our female patient, however, does not have a phenotype of X-linked LPD. Whereas the identified variant has an intermediate CADD score (i.e., 22.9), it is predicted to be benign via PolyPhen and tolerated via SIFT.

Homozygous damaging variants in *THBD* can cause aHUS. The patient does not have an aHUS phenotype and carries a heterozygous variant only.

**Patient 15**

**Homozygous Variants:** No homozygous variants left

**Heterozygous Variants:** see Table

| **GENE** | **CHR** | **POS** | **SNP_ID** | **REF** | **ALT** | **EFFECT** | **AA** | **TRANSCRIPT** | **ExAC MAF** | **READS** | **Polyphen** | **SIFT** | **CADD** |
| --- | --- | --- | --- | --- | --- | --- | --- | --- | --- | --- | --- | --- | --- |
| ***RPL5*** | **1** | **93301950** | **.** | **G** | **GT** | **SPLICE SITE DONOR** | **NA** | **ENST00000370321** | **NA** | **19** | **NA** | **NA** | **24,5** |
| *APOB* | 2 | 21232368 | . | C | G | MISSENSE | E2458Q | ENST00000233242 | < 0.01 | 39 | benign | tolerated | 9,821 |

**Abbreviations.** APOB, apolipoprotein B; NA, not annotated; RPL5, ribosomal protein L5.

**Interpretation:** The patient’s phenotype was DBA with cardiac malformations and craniofacial dysmorphism. We identified a novel heterozygous, disease-causing variant in *RPL5.* This c.527+2dupT splice site donor variant in intron 5 of *RPL5* has a moderate CADD score (i.e., 24.5). Patients with DBA and *RPL5* mutations were identified to have a higher percentage of somatic malformations (Quarel*lo, et* al 2010) and this is in-line with the phenotype in patient 15.

Homozygous damaging-variants in *APOB* can cause familial hypobetalipoproteinemia with acanthocytosis. The patient does not have this phenotype and carries a heterozygous variant. Moreover, the identified variant has a low CADD score (i.e., 9.821) and is predicted to be benign (PolyPhen) and tolerated (SIFT).

**Patient 16**

**Homozygous Variants:** see Table

| GENE | CHR | POS | EFFECT (cDNA) | TRANSCRIPT | ExAC MAF | READS | PolyPhen | SIFT | CADD |
| --- | --- | --- | --- | --- | --- | --- | --- | --- | --- |
| ***FANCA*** | **16** | **89803957 to 89883065** | **c.(522+1_522-1)_(3067+1_3067-1)del** | **ENST00000389301** | **NA** | **NA** | **NA** | **NA** | **NA** |

**Heterozygous Variants:** see Table

| **GENE** | **CHR** | **POS** | **SNP_ID** | **REF** | **ALT** | **EFFECT** | **AA** | **TRANSCRIPT** | **ExAC MAF** | **READS** | **PolyPhen** | **SIFT** | **CADD** |
| --- | --- | --- | --- | --- | --- | --- | --- | --- | --- | --- | --- | --- | --- |
| ***STEAP3*** | **2** | **120005742** | **.** | **G** | **A** | **MISSENSE** | **R337H** | **ENST00000393110** | **<0.01** | **1349** | **probably damaging** | **tolerated** | **23,9** |
| *FANCI* | 15 | 89848891 | . | C | G | MISSENSE | T1104S | ENST00000310775 | <0.01 | 741 | benign | tolerated | 12,09 |
| *RNASEH2A* | 19 | 12924219 | . | G | A | MISSENSE | R280H | ENST00000221486 | <0.01 | 700 | benign | tolerated | 11,09 |
| *SLC11A2* | 12 | 51389420 | rs368662740 | C | T | MISSENSE | E357K | ENST00000394904 | <0.01 | 660 | benign | tolerated | 3,626 |

**Abbreviations.** FANCA, Fanconi Anemia complementation group A; FANCI, Fanconi Anemia complementation group I; NA, not annotated; RNASEH2A, ribonuclease H2 subunit A; SLC11A2, solute carrier family 11 member 2; STEAP3, STEAP3 metalloreductase.

**Interpretation:** The patient’s phenotype was FA; and we identified a novel homozygous disease-causing variant in *FANCA*. This novel, large biallelic deletion variant affects exons 6 to 31 of *FANCA*; and the size is at least 56.220bp (GRCh37/hg:chr16:89.818.535-89.874.755) and maximal 60.871bp. Multiplex ligation-dependent probe amplification (MLPA) analysis (MRC-Holland MLPA; SALSA® MLPA probe mix P031-B2/P032-B2 FANCA) was performed to confirm these findings (Supplemental Figure 1); and the same variant was identified in her twin sister, who had also FA phenotype. The parents did not want to have their genes analyzed.

One other heterozygous variant, that may cause diseases was identified in *STEAP3* (anemia, hypochromic microcytic, with iron overload 2 (AHMIO2); intermediate CADD score 23.9). However, the patients have no AHMIO2 phenotypes (instead, they have macrocytic anemia) and this variant is predicted to be probably damaging (PolyPhen) and tolerated (SIFT).

Homozygous damaging variants in *FANCI, RNASEH2A* and *SLC11A2* have been reported to cause FA, Aicardi-Goutieres syndrome type 2 (AGS2) and anemia, hypochromic microcytic, with iron overload 1, respectively. Patient 16 carries heterozygous variants in these genes only, and does not have AGS2 or anemia, hypochromic microcytic, with iron overload 1 phenotypes.

**Patient 17**

**Homozygous Variants:** see Table

| **GENE** | **CHR** | **POS** | **SNP_ID** | **REF** | **ALT** | **EFFECT** | **AA** | **TRANSCRIPT** | **ExAC MAF** | **READS** | **PolyPhen** | **SIFT** | **CADD** |
| --- | --- | --- | --- | --- | --- | --- | --- | --- | --- | --- | --- | --- | --- |
| ***NHEJ1*** | **2** | **2,2E+08** | **.** | **A** | **G** | **MISSENSE** | **L79P** | **ENST00000409720** | **NA** | **1157** | **probably damaging** | **deleterious** | **31** |

**Heterozygous Variants:** see Table

| **GENE** | **CHR** | **POS** | **SNP_ID** | **REF** | **ALT** | **EFFECT** | **AA** | **TRANSCRIPT** | **ExAC MAF** | **READS** | **PolyPhen** | **SIFT** | **CADD** |
| --- | --- | --- | --- | --- | --- | --- | --- | --- | --- | --- | --- | --- | --- |
| ***CARD11*** | **7** | **2946339** | **.** | **C** | **T** | **MISSENSE** | **R1133H** | **ENST00000396946** | **<0.01** | **138** | **probably damaging** | **deleterious** | **34** |
| *CENPJ* | 13 | 25463526 | . | G | A | STOP GAINED | R1077* | ENST00000381884 | <0.01 | 105 | NA | NA | 36 |
| *RAD51C* | 17 | 56774210 | . | C | G | MISSENSE | H187Q | ENST00000337432 | NA | 315 | benign | tolerated | 3,132 |

**Abbreviations.** CARD11, caspase recruitment domain family member 11; CENPJ, centromere protein J; NA, not annotated; NHEJ1, non-homologous end joining factor 1; RAD51C, RAD51 paralog C.

**Interpretation:** The patient’s phenotype was MDS (Deletion 20q) with mild pancytopenia associated with growth retardation, microcephalus and clinodactyly. Damaging variants in *NHEJ1* were reported to cause a phenotype of immunodeficiency with microcephaly (Bu*ck, et* al 2006, Dutrann*oy, et* al 2010). We identified a novel homozygous disease-causing missense variant in *NHEJ1* (i.e., L79P). This variant has a high CADD score (i.e., 31) and is predicted to be probably damaging (PolyPhen) and deleterious (SIFT). The patient did not have severe infections but has immunodeficiency (Supplemental Table 6). The family members were not analyzed yet.

One other heterozygous variant that may cause diseases was identified in *CARD11.* Gain-of-function variants in *CARD11* can cause B-cell expansion with NF-κB and T-cell anergy (BENTA). The identified variant has a high CADD score of 34 and was predicted to be probably damaging via PolyPhen and deleterious via SIFT. The patient, however, has B-lymphopenia (Supplemental Table 6), normal IgM levels and no lymphadenopathy or splenomegaly.

Homozygous damaging variants in *CENPJ* and *RAD51C* have been reported to cause Seckel Syndrome (SS) and FA, respectively. Patient 17 carries heterozygous variants in these genes only, and does not have the phenotype of SS and has normal DEB testing.

**Supplemental Table S1.** Genes included in the Hematology panel design divided by disease group

| **Gene Name** | **Disease/Syndrome** |
| --- | --- |
| *ANK1* | Hereditary spherocytosis (HS), type 1 (SPH1) |
| *SPTB* | Hereditary eliptocytosis (HE) type 3, HS type 2 (SPH2), nonimmune hydrops fetalis (NIHF), HPP |
| *SLC4A1* | HS Type 4 (SPH4), southeast Asian ovalocytosis (SAO), HE type 4, renal tubular acidosis (RTA) |
| *SPTA1* | HE Type 2, hereditary pyropoikylocytosis (HPP), HS type 3 (SPH3) |
| *EPB42* | HS type 5 (SPH5) |
| *EPB41* | HE type 1 |
| *GYPC* | HE |
| *PIEZO1* | Dehydrated herediatry stomatocytosis (HS) w/wo pseudohyperkaliemia and/or perinatal edema (DHS), xerocytosis |
| *STOM (EPB72)* | Overhydrated hereditary stomatocytosis (OHS) |
| *RHD* | RH deficiency syndrome, stomatocytosis |
| *RHAG* | RH deficiency syndrome, stomatocytosis |
| *RHCE* | Rh autoimmune hemolytic anemia (AIHA) |
| *ABCA1* | Tangier disease |
| *ABCG5* | Sitosterolemia, phytosterolemia |
| *ABCG8* | Sitosterolemia, phytosterolemia |
| *LCAT* | Lecithin cholesterol acyltransferase deficiency |
| *MTTP* | Abetalipoproteinemia (ABL, Bassen Kornzweig syndrome) |
| *APOB* | Homozygous familial hypobetalipoproteinemia |
| *SAR1B* | Chylomicron retention disease (Anderson disease) |
| *VPS13A* | Choreo-acanthocytosis (ChAc) |
| *XK* | McLeod Syndrome (MLS) |
| *KEL* | MLS |
| *PANK2* | Pantothenate kinase associated neurodegeneration (PKAN) |
|  |  |
| **Gene Name** | **Disease/Syndrome** |
| *CYB5R3* | Methemoglobinemia due to deficiency of methemoglobin reductase (Type I) |
| *CYB5A* | Methemoglobinemia type IV |
| *G6PD* | Glucose 6 phosphate dehydrogenase (G6PD) deficiency |
| *GCLC* | Gamma-glutamyl cysteine synthetase deficiency |
| *GSS* | Glutathione synthetase deficiency |
| *GSR* | Glutathione peroxidase deficiency |
| *GPX1* | Glutathione reductase deficiency |
| *PKLR* | Pyruvat kinase deficiency (PKD) |
| *PGK1* | Phosphoglycerokinase deficiency |
| *TPI1* | Triosephosphate isomerase 1 deficiency |
| *PFKL* | Phosphofructokinase deficiency |
| *BPGM* | Bisphosphoglycerate mutase deficiency |
| *ALDOA* | Aldolase deficiency |
| *GPI* | Glucose-6-phosphate isomerase deficiency |
| *HK1* | Hexokinase deficiency |
| *HK4* | Hexokinase deficiency |
| *NT5C3A* | Pyrimidine 5 nucleotidase deficiency |
| *AK1* | Adenylate kinase deficiency |
|  |  |
| **Gene Name** | **Disease/Syndrome** |
| *HBB* | Beta thalassemia |
| *HBA1* | Hemoglobinopathies |
| *HBA2* | Hemoglobinopathies |
| *HBG1* | Hemoglobinopathies |
| *HBG2* | Hemoglobinopathies |
| *BCL11A* | Hemoglobinopathies |
| *HBS1L-MYB* | Hemoglobinopathies |
| *FCP1* | Hemoglobinopathies |
| *SLC25A38* | Autosomal recessive congenital sideroblastic anemia (CSA), Pyridoxin refractory CSA, Pearson syndrome |
| *ALAS2* | X-linked CSA |
| *GLRX5* | GLRX5-related pyridoxin-refractory autosomal recessive CSA, Pearson syndrome |
| *PUS1* | Mitochondrial myopathy, lactat acidosis, and CSA 1 |
| *SLC11A2* | Divalent metal transporter (DMT1) deficiency anemia;anemia, hypochromic, microcytic, with iron overload 1 |
| *STEAP3* | Anemia, hypochromic, microcytic, with iron overload 2 |
| *WFS1* | Wolfram Syndrome 1, diabetes mellitus, diabetes insipidus, optical atrophy, deafness (DIDMOAD) |
| *RRM2B* | Kerans-Sayre syndrome |
| *CISD2* | Wolfram syndrome 2 |
| *ABCB7* | X-linked CSA and ataxia |
| *YARS2* | Mitochondrial myopathy, lactat acidosis, and CSA 2 |
|  |  |
| **Gene Name** | **Disease/Syndrome** |
| *FANCA* | Fanconi anemia (FA) |
| *FANCB* | FA |
| *FANCC* | FA |
| *FANCD2* | FA |
| *BRCA2* | FA |
| *FANCE* | FA |
| *FANCF* | FA |
| *FANCG* | FA |
| *FANCI* | FA |
| *BRIP1* | FA |
| *PHF9* | FA |
| *FANCM* | FA |
| *PALB2* | FA |
| *RAD51C* | FA |
| *SLX4* | FA |
| *ERCC4* | FA |
| *XRCC2* | FA |
| *BLM* | Bloom syndrome |
| *LIG1* | Bloom syndrome |
| *LPIN2* | Majeed syndrome (CDA variant) |
| *DKC1* | Dyskeratosis congenital (DC) |
| *NHP2* | DC |
| *NOP10* | DC |
| *TERT* | DC |
| *TERC* | DC |
| *WRAP53* | DC |
| *RTEL1* | DC |
| *CTC1* | DC |
| *TINF2* | DC |
| *FANCD1 (BRCA1)* | FA |
| *CENPJ* | Seckel syndrome (SS) |
| *RBBP8* | SS |
| *PCNT* | SS, MVA |
| *CEP152* | SS |
| *CEP63* | SS |
| *NIN* | SS |
| *BUB1* | Premature chromatid separation (PCS), Mosaic variegated aneuploidy syndrome (MVA) type 1 |
| *BUB1B* | MVA type 1 |
| *CEP57* | MVA type 2 |
| *SMARCAL1* | Schimke immunoosseous dysplasia |
| *PTPN11* | Noonan Syndrome (NS) |
| *SOS1* | NS |
| *RAF1* | NS |
| *SHOC2* | NS |
| *CBL* | NS |
| *SIT1* | NS |
| *HRAS* | Costello Syndrome |
| *SPRED1* | Legius Syndrome |
| *BRAF* | Cardio-facio-cutaneous (CFC) syndrome |
| *NBEAL2* | Grey platelet syndrome (AR) |
| *MAP2K1* | CFC syndrome |
| *MAP2K2* | CFC syndrome |
| *NF-1* | Neurofibromatosis type 1 |
| *RPS7* | Diamond Blackfan anemia (DBA) |
| *RPS10* | DBA |
| *RPS15* | DBA |
| *RPS17* | DBA |
| *RPS19* | DBA |
| *RPS24* | DBA |
| *RPS26* | DBA |
| *RPS27A* | DBA |
| *RPS29* | DBA |
| *RPL5* | DBA |
| *RPL11* | DBA |
| *RPL15* | DBA |
| *RPL36* | DBA |
| *GATA1* | DBA, CDA variant, X-linked macrothrombocytopenia (XLMT) |
| *RUNX1* | Familial platelet disorder - AML |
| *MASTL* | MASTL-related thrombocytopenia |
| *ANKRD26* | Thrombocytopenia 2 |
| *CYCS* | Thrombocytopenia 4 |
| *MYH9* | MYH9-related thrombocytopenia (May-Hegglin, Fechtner-, Epstein-, Sebastian Syndrome) |
| *FLI1* | Paris-Trousseau syndrome |
| *GP1BA* | Bernard-Soulier syndrome type A1 (BSS), platelet type vW disease |
| *GP1BB* | BSS type B |
| *GP9* | BSS type C |
| *GFI1B* | GFI1B-related thrombocytopenia, Grey platelet syndrome (AD) |
| *FLNA* | FLN-related thrombocytopenia |
| *ACTN1* | ACTN1-related thrombocytopenia |
| *COX4I2* | CDA with exocrine pancreatic insufficiency |
| *ITGA2B* | ITGA2B-related thrombocytopenia |
| *ITG3B* | ITG3B-related thrombocytopenia |
| *TUBB1* | TUBB1-related thrombocytopenia |
| *vWF* | von Willebrand disease type 2B |
| *WAS* | Wiscott-Aldrich syndrome (WAS), X-linked thrombocytopenia (XLT) |
| *WIPF1* | WAS, XLT |
| *CDAN1* | Congenital dyserythropoeitic anemia (CDA) type I |
| *C15ORF41* | CDA type I |
| *SEC23B* | CDA type II |
| *KIF23* | CDA type III |
| *KLF1* | CDA variant |
|  |  |
| **Gene Name** | **Disease/Syndrome** |
| *CFHR3* | CFHR1/CFHR3 deficiency |
| *CFHR1* | CFHR1/CFHR3 deficiency |
| *CFH* | Factor H deficiency |
| *CFB* | Factor B deficiency |
| *CD55* | Decay-accelerating factor (CD55) deficiency |
| *CD46* | CD46 deficiency |
| *ATM* | Ataxia-telangiectasia |
| *ITGB2* | Leukocyte adhesion deficiency I |
| *FERMT3* | LAD3 deficiency |
| *SLC35C1* | Leukocyte adhesion deficiency II |
| *RAC2* | LAD with RAC2 deficiency |
| *CEBPE* | Neutrophil-specific granule deficiency |
| *TPP2* | tripeptidyl-peptidase II deficiency |
| *PGM3* | Phosphoglucomutase 3 deficiency |
| *TF* | Atransferrinemia |
| *HFE* | Hypotransferrinemia |
| *CP* | Aceruloplasminemia |
| *TMPRSS6* | Iron refractory iron deficiency anemia (IRIDA) |
| *GYPA* | Autoimmune hemolytic anemia (AIHA) |
| *CUBN* | Megaloblastic anemia 1, Imerslund Gresbeck syndrome |
| *AMN* | Megaloblastic anemia 1, Imerslund Gresbeck syndrome |
| *SLC19A2* | Thiamin responsive megaloblastic anemia (TRMA) |
| *PIGA* | Paroxysmal nocturnal hemoglobinuria (PNH) |
| *FCGR3A* | Natural killer cell deficiency |
| *RMRP* | Cartilage hair hypoplasia (CHH); Omenn syndrome |
| *UNC93B1* | UNC93B deficiency |
| *FOXP3* | X-linked immunodeficiency, polyendocrinopathy, enteropathy |
| *CD25* | IPEX/IPEX-like |
| *STAT1* | IPEX/IPEX-like |
| *MPL* | Congenital amegakaryocytic thrombocytopenia (CAMT) |
| *HOXA11* | Radioulnar synostosis with amegakaryocytic thrombocytopenia |
| *ATR* | Seckel syndrome 1 |
| *NSUN2* | Dubowitz-like syndrome |
| *GATA2* | Mononuclear cytopenias |
| *RBM8A* | TAR syndrome |
| *TCN2* | TC II deficiency; TCN2 deficiency |
| *FCGR2C* | Autoimmune thrombocytopenic purpura (AITP), systemic lupus erythematodes (SLE) |
| *TREX1* | Aicardi-Goutieres syndrome |
| *SAMHD1* | Aicardi-Goutieres syndrome |
| *RNASEH2C* | Aicardi-Goutieres syndrome |
| *RNASEH2B* | Aicardi-Goutieres syndrome |
| *RNASEH2A* | Aicardi-Goutieres syndrome |
| *THBD* | Atypical HUS (Hemolytic Uremic Syndrome) |
| *RECQL4* | Rothmund-Thomson syndrome with immunodeficiency |
| *IRF8* | IRF8 deficiency |
|  |  |
| **Gene Name** | **Disease/Syndrome** |
| *USB1* | Poikiloderma with neutropenia |
| *ELANE* | Congenital neutropenia (CN) |
| *HAX1* | (CN) |
| *G6PC3* | (CN) |
| *JAGN1* | (CN) |
| *TYK2* | (CN) |
| *WASP* | (CN) |
| *GCSFR* | (CN) |
| *TCIRG1* | (CN) |
| *SBDS* | Shwachman-Diamond syndrome |
| *LYST/CHS* | Congenital neutropenia w/ hypopigmentation |
| *AP3B1* | Hermansky-Pudlak syndrome 2 |
| *AP3D1* | Congenital neutropenia w/ hypopigmentation |
| *AP3S1* | Congenital neutropenia w/ hypopigmentation |
| *AP3M1* | Congenital neutropenia w/ hypopigmentation |
| *CD1b* | Congenital neutropenia w/ hypopigmentation |
| *MAPBPIP* | Congenital neutropenia w/ hypopigmentation |
| *CXCR4* | Complex disorder with congenital neutropenia |
| *G4.5/TAZ* | Complex disorder with congenital neutropenia; Barth syndrome |
| *VPS13B/COH* | Complex disorder with congenital neutropenia |
| *GFI1* | Severe congenital neutropenia, autosomal dominant 2; SCN2 |
| *SLC37A4* | Glycogen storage disease 1b; Glycogen storage disease 1c |
| *ROBLD3* | Endosomal adaptor protein p14 deficiency |
| *CSF3R* | CN |
|  |  |
| **Gene Name** | **Disease/Syndrome** |
| *TACI* | Hypogammaglobulinemia with low serum IgG, IgM, and IgA, and recurrent infections, including otitis media, respiratory tract infections, and gastrointestinal tract infections. |
| *BAFFR* | Low levels of serum IgG and IgM, but normal serum and mucosal IgA. T cell-dependent antigen response to vaccine, but defective T cell-independent humoral responses. No BAFFR surface expression in B cells |
| *CD19* | Susceptibility to infection, hypogammaglobulinemia, and normal numbers of mature B cells in blood, indicating a B-cell antibody-deficient immunodeficiency disorder |
| *CD20* | Recurrent respiratory infections, persistent hypogammaglobulinemia, reduced circulating memory B cells, and complete lack of surface CD20 on B cells. |
| *CD21* | Recurrent respiratory infections, splenomegaly, hypogammaglobulinemia, absence of CD21 antigen. Reduced binding to a C3d-containing immune complex |
| *CD81* | Recurrent respiratory tract infections, hepatomegaly and thrombocytopenia associated with antiplatelet antibodies. Hypogammaglobulinemia. Lack of CD19 and CD81 surface expression in B-cells |
| *ICOS* | Antibody deficiency, hypogammaglobulinemia, recurrent bacterial infections, and an inability to mount an antibody response to antigen. |
| *LRRC8A* | Non-Bruton type autosomal dominant agammaglobulinemia |
| *LRBA* | Early childhood onset of recurrent infections, particularly respiratory infections, and also develop variable autoimmune disorders, including idiopathic thrombocytopenic purpura, autoimmune hemolytic anemia, and inflammatory bowel disease. |
| *UNG* | Increased serum IgM concentrations associated with low or absent serum IgG, IgA, and IgE concentrations, indicating a defect in the class-switch recombination (CSR) process. |
| *SP110* | CID; VODI; Hepatic veno-occlusive disease with immunodeficiency syndrome; Hepatic veno- occlusive disease-immunodeficiency |
|  |  |
| **Gene Name** | **Disease/Syndrome** |
| *PTPRC* | CD45 deficiency. T cell-negative (T-), B cell-positive (B+), natural killer cell-positive (NK+) |
| *RAG1* | (S)CID T-, B-, NK+ |
| *RAG2* | (S)CID T-, B-, NK+ |
| *RFX5* | (S)CID. Bare lymphocyte syndrome type II |
| *RFXANK* | (S)CID. MHC class II deficiency, complementation group B |
| *RFXAP* | (S)CID. Bare lymphocyte syndrome, type II |
| *STIM1* | Ca2+ channel deficiency; Immune dysfunction with T-cell inactivation due to calcium entry defect 2 |
| *TBX1* | DiGeorge syndrome. |
| *ZAP70* | ZAP70 deficiency. Selective T-cell defect |
| *PNP* | (S)CID. Decreased T-cell function |
| *NME1* | (S)CID |
| *STAT5B* | (S)CID/ immune dysregulation, polyendocrinopathy, enteropathy, X-linked (IPEX)-like |
| *CD40L* | X-linked Hyper-IgM syndrome; CD40 ligand deficiency |
| *CD40* | CD40 deficiency; Autosomal recessive hyper-IgM syndrome |
| *SH2D1A* | (S)CID/XLP Lymphoproliferative syndrome |
| *STK4* | (S)CID. T-cell immunodeficiency, recurrent infections, autoimmunity, and cardiac malformations |
| *TCRA* | (S)CID. T-cell receptor-alpha/beta deficiency |
| *LCK* | p56 Lck deficiency. Lymphopenia and hypogammaglobulinemia |
| *PI3KD* | (S)CID |
| *CTLA4* | (S)CID. Autoimmune lymphoproliferative syndrome, type V |
| *ITK* | ITK deficiency. Lymphoproliferative syndrome 1 |
| *TTC7A* | T and B lymphopenia and gastrointestinal defects |
| *DOCK8* | Lymphopenia. Hyper-IgE recurrent infection syndrome |
| *UNC119* | CD4 lymphopenia. Reduction in the CD4 T-lymphocyte count |
| *CD3G* | CD3 gamma deficiency. T-cell lymphopenia. |
| *CD3E* | CD3 epsilon deficiency. T cell-negative, B cell-positive, natural killer (NK) cell-positive phenotype. |
| *CD3D* | CD3 delta deficiency. T cell-negative, B cell-positive, natural killer (NK) cell-positive phenotype |
| *ORAI1* | (S)CID: early onset of recurrent infections due to defective T-cell activation |
| *ADA* | Adenosine deaminase deficiency: T cell-negative (T-), B cell-negative (B-), natural killer cell- negative (NK-) severe combined immunodeficiency |
| *AK2* | Reticular dysgenesis, congenital agranulocytosis, lymphopenia, and lymphoid and thymic hypoplasia with absent cellular and humoral immunity functions. |
| *CD247* | CD3Zeta deficiency. Low T-cells, eosinophilia. |
| *CD8A* | (S)CID: absence of CD8-positive T cells, both CD3+ and CD3-. Normal CD4, B cells and NK cells. |
| *CIITA* | CIITA, MHC II transactivating protein deficiency; MHC class II deficiency. Bare lymphocyte syndrome |
| *CORO1A* | (S)CID: decreased numbers of lymphocytes, poor T-cell function with decreased proliferative response and lack of helper T-cell function for antibody isotype switching, and low immunoglobulins. B and NK cells were normal. |
| *DCLRE1C* | (S)CID: T cell-negative (T-), B cell-negative (B-), natural killer cell-positive (NK+) severe combined immunodeficiency with sensitivity to ionizing radiation |
| *FOXN1* | (S)CID: T-cell immunodeficiency, congenital alopecia, and nail dystrophy |
| *IKZF1* | Ikaros deficiency. recurrent bacterial infections, mainly affecting the respiratory tract, and associated with hypogammaglobulinemia and decreased numbers of B cells |
| *IL2RG* | Combined immunodeficiency, X-linked, T-, B+, NK- SCID |
| *IL7R* | (S)CID: Severe combined immunodeficiency, T-cell negative, B-cell/natural killer cell-positive type |
| *JAK3* | (S)CID autosomal recessive, T-negative/B-positive type |
| *LIG4* | LIG4 syndrome; DNA ligase IV deficiency; Omenn syndrome; RS-SCID. Unusual facial features, microcephaly, growth and/or developmental delay, pancytopenia, and various skin abnormalities |
| *NHEJ1* | Cernunnos deficiency. SCID with microcephaly, growth retardation, and sensitivity to ionizing radiation |
| *NBN* | Nijmegen breakage syndrome. Microcephaly, growth retardation, immunodeficiency, and predisposition to cancer. |
| *PRKDC* | (S)CID. Lower respiratory tract infections, as well as a large oral aphthous lesion. B and T cells were virtually absent from peripheral blood, but NK cells were normal. Bone marrow analysis showed a complete block in B-cell differentiation. |
|  |  |
| **Gene Name** | **Disease/Syndrome** |
| *CASP10* | Autoimmune lymphoproliferative syndrome (ALPS), type II |
| *CASP8* | ALPS, type IIB |
| *FADD* | Recurrent infections with encephalopathy, hepatic dysfunction and cardiovasuclar malformations |
| *CARD11* | Block in B-cell differentiation with increased numbers of transitional B cells and hypogammaglobulinemia, as well as decreased numbers of regulatory T cells and defects in T-cell function |
| *PRKCD* | ALPS, type III |
| *KRAS* | RAS-associated autoimmune leukoproliferative disease |
| *NRAS* | ALPS type IV |
| *FASLG* | ALPS, type 1B |
| *FAS* | ALPS, type Ia |
|  |  |
| **Gene Name** | **Disease/Syndrome** |
| *XIAP/BIRC4* | X-linked lymphoproliferative syndrome (XLP) |
| *PRF1* | FHL w/ or w/o hypopigmentation |
| *UNC13D* | FHL w/ or w/o hypopigmentation |
| *STX11* | FHL w/ or w/o hypopigmentation |
| *STXBP2* | FHL w/ or w/o hypopigmentation |
| *LYST* | Chediak-Higashi syndrome |
| *AP3B4* | FHL w/ or w/o hypopigmentation |
| *RAB27A* | FHL w/ or w/o hypopigmentation |

**Supplemental Table S2. Pertinent data of the 11** patients with anemia.

| **Patient ID (age/years, ethnicity)** | **Hb level**  **(nadir) g/dl** | **MCV (fl)** | **Reticulocytes (%)** | **RDW (%)** | **Total bilirubin**  **(mg/dl)** | **LDH**  **(U/l)** | **Haptoglobin**  **(mg/dl)** | **Eosin-5-maleimide binding** | **Hb-HPLC** | **RBC enzyme levels/ folate and vitB12** | **Morphology:**  **RBC/bone marrow** | **Diagnosis before / after genetic testing (Gene affected)** | **Clinical findings** | **RBC transfusion(s) / other therapy** |
| --- | --- | --- | --- | --- | --- | --- | --- | --- | --- | --- | --- | --- | --- | --- |
| **P1 (13.7, European)** | 11 (3.4) | 82 | 13 | 20 | 5.8 | 284 | 0 | reduced | normal | normal / normal | Anisocytosis, spherocytes / n.d. | HS / HS type 2 (*SPTB*) | Moderate CHA, jaundice, splenomegaly, gall stones | 3x / none |
| **P2 (8, European)** | 9.2 (6.6) | 77 | 10.6 | 23.3 | 3.3 | 286 | 0 | reduced | n.d. | n.d. / n.d. | Anisocytosis, spherocytes / n.d. | HS / HS type 2 (*SPTB*) | Moderate CHA, jaundice, splenomegaly, gall stones; marked improved after surgery | 3x / subtotal splenectomy and cholecystectomy |
| **P3 (13.6, European)** | 13.3 (5.1) | 87 | 10.3 | 17.7 | 6.9 | 317 | < 10 | n.d.; glycerol lysis and kryohemolysis tests were pathologic | n.d. | n.d. / n.d. | Anisocytosis, spherocytes / n.d. | HS / HS type 1 (*ANK1*) | Moderate CHA, jaundice, splenomegaly, gall stones; marked improved after surgery | 17x / subtotal splenectomy and cholecystectomy |
| **P4 (1, European)** | 9.5 (7.9) | 74 | 7.2 | 17 | 0.8 | 245 | < 10 | reduced | normal | normal / n.d. | Anisocytosis, spherocytes / n.d. | HS / HS type 1 (*ANK1*) | Moderate CHA, jaundice, splenomegaly | 7x (none since age 6 months) |
| **P5 (2.7, European)** | 9.2 (4.9) | 80 | 10.4 | 21.3 | 0.9 | 239 | <10 | reduced | n.d. | normal /n.d. | Anisocytosis, spherocytes / n.d. | HS / HS type 1 (*ANK1*) | Moderate CHA, jaundice, splenomegaly | 12x |
| **P6 (4.6, Turkish)** | 9.2 (5.1) | 97 | 14 | 18.8 | 3.1 | 904 | < 10 | normal | HbA 94.6%  HbA2 2.9%  HbF 2.5% | PK activity in lower normal range (but reticulocytosis) / normal | Anisocytosis, macrocytes / dyserythropoiesis (3% bi-nucleated) | CDA / PKD (*PKLR*) | Severe CHA, jaundice, splenomegaly | 23x / folinic acid |
| **P7 (19.2, European)** | 14.1 (12.8) | 106 | 10.3 | 17 | 9.2 | 144 | < 10 | normal | normal | normal / normal | Target cells, dessicytes, / n.t. | ‚Familial jaundice‘ / HX (*PIEZO1*) | Mild CHA, severe jaundice, splenomegaly, gall stones | Cholecystectomy planned |
| **P8 (10.1, Turkish)** | 10.9 (5.1) | 100 | 10.3 | 16.3 | 7.2 | 289 | < 10 | normal | normal | normal / normal | Target cells, dessicytes, (Fig. 1B) / increased erythropoiesis | CHA / HX (*PIEZO1*) | Mild CHA, severe jaundice, splenomegaly | 1x (Parvo virus infection) |
| **P9 (3.4, European)** | 9.0 (6.0) | 87 | 2.3 | 27.7 | 1.7 | 246 | < 10 | normal | HbA 94.4%  HbA2 3.7%  HbF 1.9% | normal / normal | Anisocytosis, poikilocytosis (Fig. 1D) / erythroid hyperplasia with internuclear bridges between erythroblasts (Fig. 1E) | CHA / CDA Type 1 (*CDAN1*) | Moderate CHA, splenomegaly | 12x (none since 14 months of age) /interferon therapy was refused by parents |
| **P10 (17.9, European)** | 11.2 (no data) | 99 | 3.4 | 26.6 | 2.6 | 379 | <10 | normal | HbA 94.5%  HbA2 3.5%  HbF 2.8% | normal / normal | Anisocytosis, poikilocytosis / erythroid hyperplasia with internuclear bridges between erythroblasts | CHA / CDA Type 1 (*CDAN1*) | Moderate CHA, splenomegaly, cardiac defects, clinodactyly, Chiari malformation type 1, syringomyelia | Ttransfusions during first year of life / interferon and iron chelation therapy planned |
| **P11 (1.2, European)** | 5.4 (5.4) | 101 | 0.4 | 18.3 | 0.4 | 346 | 66 | n.d. | HbA 84.7%  HbA2 2.0%  HbF 13.3% (age 14 months) | normal / normal | Macrocytic anemia, reticulocytopenia / normocellular bone marrow with lack of erythroblasts | DBA / DBA (*RPS29*) | Severe anemia, no skeletal or cardiac anomalies | Initiation of transfusions, treated at other centre |

Hemoglobin, MCV, RDW, and reticulocytes were measured via Advia 2120i Hematology (Siemens) cell counter. Morphology analysis was performed on May-Grünwald-Giemsa-stained blood and bone marrow films. Eosin-5-maleimide binding was performed as reported (Ki*ng, et* al 2000). RBC enzyme activities were measured on a Cobas Mira Plus (Hoffmann-La Roche, Basel Switzerland) according to the method proposed by Beutler (Beutler, 1984).

**Abbreviations:** ANK1, Ankyrin 1; CDA1, congenital dyserythropoietic anemia type 1; CDAN1, codanin 1; CHA, chronic hemolytic anemia; DBA, Diamond Blackfan anemia; Hb, hemoglobin; HPLC, high-performance liquid chromatography; HS, hereditary spherocytosis; HX, hereditary xerocytosis; n.d., not done; MCV, mean corpuscular volume; PIEZO1, piezo type mechanosensitive ion channel component 1; PKD, pyruvate kinase deficiency; PKLR, pyruvate kinase, liver and RBC; RBC, red blood cell; RDW, red cell distribution width; RPL5, ribosomal protein L5; RPS29, ribosomal protein S29; SPTB, spectrin beta, erythrocytic, vitB12, vitamin B12

**Supplemental Table S3. Pertinent data of the two** patients with thrombocytopenia.

| **Patient ID (age/years, ethnicity)** | **Platelets**  **(nadir) 10^9^/L** | **MPV (fl)** | **Morphology:**  **Peripheral blood/bone marrow** | **Additional investigations** | **Diagnosis before / after genetic testing (Gene affected)** | **Clinical findings** | **Thrombocyte transfusion(s) / other therapy** |
| --- | --- | --- | --- | --- | --- | --- | --- |
| **P12 (8.8, European)** | 100  (30) | 8 | normal / n.d. | Functional thrombocyte analyses and broad family screening planned to be performed; FISH analysis for MDS and flow-cytometry MRD analysis for leukemia revealed no evidence for MDS-typical aberrations or leukemia cells | cITP / RUNX1 associated thrombocytopenia (*RUNX1*) | Mild thrombocytopenia since birth, aggravation without bleeding signs during infections; no increased bleeding tendency reported | none |
| **P13 (0.9, European)** | 6 (3) ^†^ | 7^†^ | Giant platelets (Fig. 1G) / normocellular | Immunostaining of non-muscular myosin IIA (NMM IIA) in leucocytes: very small inclusion bodies detected | Macrothrombocytopenia / MYH9-related disease (*MYH9*) | Only a few petechiae and bruising; mucous membrane bleeding once during viral infectious disease | 6x (5x before diagnosis) / tranexamic acid and/or desmopressin if bleeding |

Platelet count and volume was measured by Advia 2120i Hematology (Siemens) cell counter. Morphology analysis was performed on May-Grünwald-Giemsa-stained blood and bone marrow films. ^†^In the cell counter the giant platelets were not counted as platelets and therefore the degree of thrombocytopenia was overestimated and the MPV was underestimated. Via optical microscopy analyses on May-Grünwald-Giemsa-stained blood films the thrombocyte diameter was measured as being 7.3 µm, and the thrombocyte count was 30 x10^9^/L (counter analysis 3 x10^9^/L).

**Abbreviations:** cITP, chronic immunthrombocytopenia; FISH, fluorescent in-situ hybridization; MDS, myelodysplastic syndrome; MPV, mean platelet volume; MRD-Minimal residual disease; MYH9, myosin heavy chain 9; RUNX1, runt related transcription factor 1.

**Supplemental Table S**4. Pertinent data of the patient with leukopenia.

| **Patient ID (age/years, ethnicity)** | **WBC**  **(nadir) 10^9^/L** | **ANC (nadir) 10^9^/L** | **Lymphocytes (nadir) 10^9^/L** | **Bone marrow morphology** | **Additional investigations** | **Diagnosis before / after genetic testing (Gene affected)** | **Clinical findings** | **Clinical consequences** |
| --- | --- | --- | --- | --- | --- | --- | --- | --- |
| **P14 (17.8, European)** | 2.14 (1.24) | 1.56 (0.11) | CD19(+): 0.1 (0.07)  CD3(+): 0.74 (0.56) | Normal trilinear maturation, increased number of monocytes | FISH analysis for MDS and flow-cytometry MRD analysis for leukemia revealed no evidence for MDS-typical aberrations or leukemia cells | Immune mediated neutropenia / GATA2 associated neutropenia (*GATA2*) | Recurrent aphthous lesions in the mouth associated with fever, leukopenia | Surveillance for GATA2 associated complications |

WBC count and ANC were measured by Advia 2120i Hematology (Siemens) cell counter. Morphology analysis was performed on May-Grünwald-Giemsa-stained blood and bone marrow films.

**Abbreviations:** ANC, absolute neutrophil count; CD3(+), CD3 positive T-lymphocytes; CD19(+), CD19 positive B-lymphocytes; GATA2, GATA binding protein 2; FISH, fluorescent in-situ hybridization; MDS, myelodysplastic syndrome; MRD, minimal residual disease; WBC, white blood cells

**Supplemental Table S**5. Pertinent data of the patient with bicytopenia.

| **Patient ID (age/years, ethnicity)** | **Hb level**  **(nadir) g/dl** | **Reticulocytes (%)** | **WBC**  **(nadir) 10^9^/L** | **ANC (nadir) 10^9^/L** | **Additional investigations** | **Bone marrow morphology** | **Diagnosis before / after genetic testing (Gene affected)** | **Clinical findings** | **RBC transfusion(s) / other therapy** |
| --- | --- | --- | --- | --- | --- | --- | --- | --- | --- |
| **P15 (13, European)** | transfused | 0.4 | 2.5 (0.95) | 1.1 (0.39) | eADA reduced; FISH analysis for MDS and flow-cytometry MRD analysis for leukemia revealed no evidence for MDS-typical aberrations or leukemia cells; normal chromosome fragility | Hypocellular with trilinear maturation | DBA / DBA (*RPL5*) | Anemia, iron overload, growth retardation, cardiac defects | Currently regular RBC transfusions / pause of steroid therapy during puberty (steroid responder); iron chelation |

Hb, reticulocytes, WBC count and ANC were measured by Advia 2120i Hematology (Siemens) cell counter. Morphology analysis was performed on May-Grünwald-Giemsa-stained blood and bone marrow films.

**Abbreviations:** ANC, absolute neutrophil count; DBA, Diamond Blackfan anemia; DEB, eADA, erythrocyte adenosine deaminase; FISH, fluorescent in-situ hybridization; Hb, hemoglobin; MDS, myelodysplastic syndrome; MRD-Minimal residual disease; RBC, red blood cell; RPL5, ribosomal protein L5; WBC, white blood cells

**Supplemental Table S6. Pertinent data of the two** patients with pancytopenia.

| **Patient ID (age/years, ethnicity)** | **Hb level**  **(nadir) g/dl** | **MCV (fl)** | **Platelets**  **(nadir) 10^9^/L** | **WBC**  **(nadir) 10^9^/L** | **ANC (nadir) 10^9^/L** | **Lymphocytes 10^9^/L** | **Chromosome fragility testing with DEB** | **FISH analysis^†^** | **Bone marrow morphology** | **Diagnosis before / after genetic testing (Gene affected)** | **Clinical findings** | **Clinical consequences** |
| --- | --- | --- | --- | --- | --- | --- | --- | --- | --- | --- | --- | --- |
| **P16 (8.3, Turkish)** | 11.8 (10) | 102 | 81 (45) | 4.2 (3.6) | 1.3 (1) | CD3(+): 1.58  CD19(+): 0.55 | pathologic | No pathologic clones detected | Cellularity slightly reduced | FA / FA (*FANCA*) | Mild pancytopenia, epicanthus, broad nasal base, cardiac defect | Tumor surveillance |
| **P17 (21.4, Pakistan)** | 13 (8.2) | 98 | 94 (45) | 3.46 (1.68) | 1.7 (0.53) | CD3(+): 0.82  CD19(+): 0.02 | normal | 68% del(20)(q11); initially monosomy 7 clone, which was replaced by del 20q clone | Initially dysplastic (RCC) but over time normalized | MDS / CID with MDS (*NHEJ1*) | Mild stable pancytopenia, immunodeficiency, microcephalus, growth retardation, clinodactyly | Consider HSCT |

Hb, MCV, WBC count and ANC were measured by Advia 2120i Hematology (Siemens) cell counter. Morphology analysis was performed on May-Grünwald-Giemsa-stained blood and bone marrow films.

**Abbreviations:** ANC, absolute neutrophil count, CD3(+), CD3 positive T-lymphocytes; CD19(+), CD19 positive B-lymphocytes; CID, combined immunodeficiency; DEB, diepoxybutane; FA, Fanconi anemia; FANCA, Fanconi Anemia Complementation Group A; FISH, fluorescent in-situ hybridization; Hb, hemoglobin; HSCT, hematopoietic stem cell transplantation; MCV, mean corpuscular volume; MDS, myelodysplastic syndrome; MRD-Minimal residual disease; RCC, refractory cytopenia of childhood; WBC, white blood cells

**Supplemental** **Figure S1:** MLPA results with the FANCA kits P031-B2 (left) and P032-B2 (right). Results from normal controls (peak ratio 1 corresponding to 2 copies) and the twin girls harboring a biallelic deletion of exons 6-31 of the *FANCA* gene (peak ratio 0) are shown.


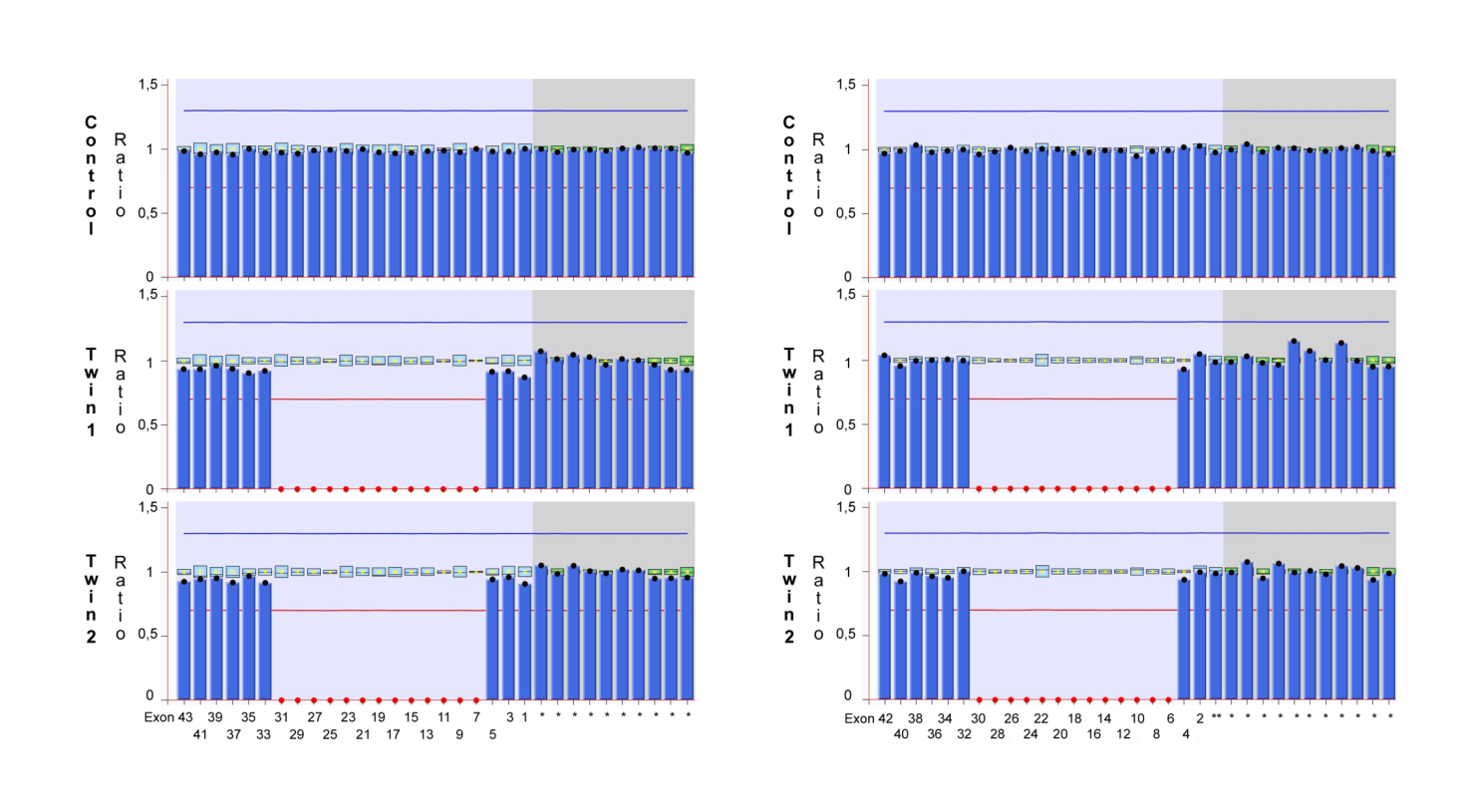


**Supporting References**

Adzhubei, I.A., Schmidt, S., Peshkin, L., Ramensky, V.E., Gerasimova, A., Bork, P., Kondrashov, A.S. & Sunyaev, S.R. (2010) A method and server for predicting damaging missense mutations. *Nature Methods,* **7,** 248-249.

Albuisson, J., Murthy, S.E., Bandell, M., Coste, B., Louis-Dit-Picard, H., Mathur, J., Feneant-Thibault, M., Tertian, G., de Jaureguiberry, J.P., Syfuss, P.Y., Cahalan, S., Garcon, L., Toutain, F., Simon Rohrlich, P., Delaunay, J., Picard, V., Jeunemaitre, X. & Patapoutian, A. (2013) Dehydrated hereditary stomatocytosis linked to gain-of-function mutations in mechanically activated PIEZO1 ion channels. *Nature Communications,* **4,** 1884.

An, X. & Mohandas, N. (2008) Disorders of red cell membrane. *British Journal of Haematology,* **141,** 367-375.

Baronciani, L., Magalhaes, I.Q., Mahoney, D.H., Jr., Westwood, B., Adekile, A.D., Lappin, T.R. & Beutler, E. (1995) Study of the molecular defects in pyruvate kinase deficient patients affected by nonspherocytic hemolytic anemia. *Blood Cells, Molecules and Diseases,* **21,** 49-55.

Beutler E, editor. (1984) Red cell metabolism: A manual of biochemical methods (3rd edition), Orlando: Grune & Stratton, Inc.

Buck, D., Malivert, L., de Chasseval, R., Barraud, A., Fondaneche, M.C., Sanal, O., Plebani, A., Stephan, J.L., Hufnagel, M., le Deist, F., Fischer, A., Durandy, A., de Villartay, J.P. & Revy, P. (2006) Cernunnos, a novel nonhomologous end-joining factor, is mutated in human immunodeficiency with microcephaly. *Cell,* **124,** 287-299.

Dgany, O., Avidan, N., Delaunay, J., Krasnov, T., Shalmon, L., Shalev, H., Eidelitz-Markus, T., Kapelushnik, J., Cattan, D., Pariente, A., Tulliez, M., Cretien, A., Schischmanoff, P.O., Iolascon, A., Fibach, E., Koren, A., Rossler, J., Le Merrer, M., Yaniv, I., Zaizov, R., Ben-Asher, E., Olender, T., Lancet, D., Beckmann, J.S. & Tamary, H. (2002) Congenital dyserythropoietic anemia type I is caused by mutations in codanin-1. *The American Journal of Human Genetics,* **71,** 1467-1474.

Dutrannoy, V., Demuth, I., Baumann, U., Schindler, D., Konrat, K., Neitzel, H., Gillessen-Kaesbach, G., Radszewski, J., Rothe, S., Schellenberger, M.T., Nurnberg, G., Nurnberg, P., Teik, K.W., Nallusamy, R., Reis, A., Sperling, K., Digweed, M. & Varon, R. (2010) Clinical variability and novel mutations in the NHEJ1 gene in patients with a Nijmegen breakage syndrome-like phenotype. *Human Mutation,* **31,** 1059-1068.

Holme, H., Hossain, U., Kirwan, M., Walne, A., Vulliamy, T. & Dokal, I. (2012) Marked genetic heterogeneity in familial myelodysplasia/acute myeloid leukaemia. *British Journal of Haematology,* **158,** 242-248.

Kanagal-Shamanna, R., Loghavi, S., DiNardo, C.D., Medeiros, L.J., Garcia-Manero, G., Jabbour, E., Routbort, M.J., Luthra, R., Bueso-Ramos, C.E. & Khoury, J.D. (2017) Bone marrow pathologic abnormalities in familial platelet disorder with propensity for myeloid malignancy and germline RUNX1 mutation. *Haematologica,* **102,** 1661-1670.

King, M.J., Behrens, J., Rogers, C., Flynn, C., Greenwood, D. & Chambers, K. (2000) Rapid flow cytometric test for the diagnosis of membrane cytoskeleton-associated haemolytic anaemia. *British Journal of Haematology,* **111,** 924-933.

Kircher, M., Witten, D.M., Jain, P., O'Roak, B.J., Cooper, G.M. & Shendure, J. (2014) A general framework for estimating the relative pathogenicity of human genetic variants. *Nature Genetics,* **46,** 310-315.

Liszewski, M.K. & Atkinson, J.P. (2015) Complement regulator CD46: genetic variants and disease associations. *Human Genomics,* **9,** 7.

Mirabello, L., Macari, E.R., Jessop, L., Ellis, S.R., Myers, T., Giri, N., Taylor, A.M., McGrath, K.E., Humphries, J.M., Ballew, B.J., Yeager, M., Boland, J.F., He, J., Hicks, B.D., Burdett, L., Alter, B.P., Zon, L. & Savage, S.A. (2014) Whole-exome sequencing and functional studies identify RPS29 as a novel gene mutated in multicase Diamond-Blackfan anemia families. *Blood,* **124,** 24-32.

Ng, P.C. & Henikoff, S. (2003) SIFT: Predicting amino acid changes that affect protein function. *Nucleic Acids Research,* **31,** 3812-3814.

Noris, P., Biino, G., Pecci, A., Civaschi, E., Savoia, A., Seri, M., Melazzini, F., Loffredo, G., Russo, G., Bozzi, V., Notarangelo, L.D., Gresele, P., Heller, P.G., Pujol-Moix, N., Kunishima, S., Cattaneo, M., Bussel, J., De Candia, E., Cagioni, C., Ramenghi, U., Barozzi, S., Fabris, F. & Balduini, C.L. (2014) Platelet diameters in inherited thrombocytopenias: analysis of 376 patients with all known disorders. *Blood,* **124,** e4-e10.

Pecci, A., Klersy, C., Gresele, P., Lee, K.J., De Rocco, D., Bozzi, V., Russo, G., Heller, P.G., Loffredo, G., Ballmaier, M., Fabris, F., Beggiato, E., Kahr, W.H., Pujol-Moix, N., Platokouki, H., Van Geet, C., Noris, P., Yerram, P., Hermans, C., Gerber, B., Economou, M., De Groot, M., Zieger, B., De Candia, E., Fraticelli, V., Kersseboom, R., Piccoli, G.B., Zimmermann, S., Fierro, T., Glembotsky, A.C., Vianello, F., Zaninetti, C., Nicchia, E., Guthner, C., Baronci, C., Seri, M., Knight, P.J., Balduini, C.L. & Savoia, A. (2014) MYH9-related disease: a novel prognostic model to predict the clinical evolution of the disease based on genotype-phenotype correlations. *Human Mutation,* **35,** 236-247.

Quarello, P., Garelli, E., Carando, A., Brusco, A., Calabrese, R., Dufour, C., Longoni, D., Misuraca, A., Vinti, L., Aspesi, A., Biondini, L., Loreni, F., Dianzani, I. & Ramenghi, U. (2010) Diamond-Blackfan anemia: genotype-phenotype correlations in Italian patients with RPL5 and RPL11 mutations. *Haematologica,* **95,** 206-213.

Unal, S. & Gumruk, F. (2015) Molecular Analyses of Pyruvate Kinase Deficient Turkish Patients from a Single Center. *Pediatr Hematol Oncol,* **32,** 354-361.

Zarychanski, R., Schulz, V.P., Houston, B.L., Maksimova, Y., Houston, D.S., Smith, B., Rinehart, J. & Gallagher, P.G. (2012) Mutations in the mechanotransduction protein PIEZO1 are associated with hereditary xerocytosis. *Blood,* **120,** 1908-1915.
